# A Sleeping Opportunity Does Not Restore Hippocampal Alterations Induced by 10 Days of Sleep Restriction in Rats

**DOI:** 10.1007/s11064-025-04561-1

**Published:** 2025-09-29

**Authors:** Jesús Enrique García-Aviles, Jessica J. Avilez-Avilez, Josué Sánchez-Hernández, Camila Patlán-Márquez, Javier Rodríguez-Alpízar, Fernanda Michell Becerril-Mercado, Adriana Jiménez, Natalí N. Guerrero-Vargas, Jean-Pascal Morin, Melissa Rodríguez-García, Joaquín Manjarrez-Marmolejo, Beatriz Gómez-González, Rosalinda Guevara-Guzmán, Mara A. Guzmán-Ruiz

**Affiliations:** 1 Departamento de Fisiología, Facultad de Medicina, Universidad Nacional Autónoma de México (UNAM), Mexico City, Mexico; 2 Área de neurociencias, Departamento de biología de la reproducción, CBS, Universidad Autónoma Metropolitana, Unidad Iztapalapa, Mexico City, Mexico; 3 Posgrado en Biología Experimental, Universidad Autónoma Metropolitana, Unidad Iztapalapa, Mexico City, Mexico; 4 División de Investigación, Hospital Juárez de México, Mexico City, Mexico; 5 Departamento de Anatomía, Facultad de Medicina, Universidad Nacional Autónoma de México (UNAM), Mexico City, Mexico; 6 Laboratorio de Fisiología de la Formación Reticular, Instituto Nacional de Neurología y Neurocirugía, Mexico City, Mexico; 7 Universidad Nacional Autónoma de México (UNAM), Av. Universidad 3004, Copilco Universidad, Delegación Coyoacán, 04510 Ciudad de México, CDMX Mexico

**Keywords:** Hippocampus, Sleep restriction, Local-field potentials, Microglia, ERK

## Abstract

Sleep loss has been implicated in age-related cognitive decline. Experimental sleep restriction (SR) alters the physiology of multiple brain regions and increases blood–brain barrier (BBB) permeability. Among these regions, the hippocampus of both humans and rodents shows alterations that endure longer than in other areas such as the basal ganglia and hypothalamus. In the present study, adult male rats were subjected to 10 days of SR using the modified multiple platform method (MMPM). Immediately after restriction, SR animals exhibited increased IBA-1 immunoreactivity (IR) and cell number, consistent with microglial activation; these morphological changes persisted after a 4 h recovery period. Synaptophysin (Syn) expression was significantly reduced after SR and remained decreased following rest, while the pERK/ERK ratio was significantly increased by the end of the recovery window. These molecular alterations were accompanied by disrupted hippocampal local field potentials (LFPs), characterized by increased alpha and beta activity and reduced delta and theta power. Importantly, SR rats showed impaired short-term memory in the novel object and object location recognition tests after the recovery period. Together, these findings demonstrate that subchronic SR induces persistent microglial and synaptic alterations and abnormal ERK signaling that remain after short rest, correlating with hippocampal network dysfunction and memory impairment.

**Supplementary Information:**

The online version contains supplementary material available at 10.1007/s11064-025-04561-1.

## Introduction

The total or partial loss of sleep, defined as sleep restriction (SR), represents a health problem that increases the risk for developing conditions such as overweight, obesity, infectious and cardiovascular diseases, diabetes and hypertension [1]. Many processes aimed at maintaining homeostasis take place preferentially during sleep. For example, central nervous system (CNS) debris clearance, and several synaptic plasticity processes involved in memory consolidation and cognition take place during specific phases of the sleep cycle [2–4]. In addition, there is growing evidence suggesting that SR may be a risk factor for the development of neurodegenerative diseases [5]. Human studies indicate that short sleep duration (< 7 h per night) is associated with a higher incidence of neurodegenerative conditions [6, 7]. The hippocampus (Hc) is sensitive to SR and sleep deprivation with changes in cellular signaling, protein synthesis, and neuronal connectivity following sleep loss [8]. Sleep loss in rodents induces a decrease in hippocampal synaptic proteins, postsynaptic protein 95 (PSD-95) and synaptophysin (Syn), as well as a decrease in the phosphorylation levels of extracellular signal-regulated kinase (ERK), a kinase involved in memory formation, consolidation, and retrieval [9–11]. Sleep loss also increases the expression of the macrophage marker ionized calcium-binding adaptor molecule 1 (IBA-1) and promotes the expression of inflammatory cytokines in the CNS [8, 12–17].

Sleep loss induces hippocampal long-lasting effects. In humans, one night of sleep deprivation impairs hippocampal dependent memory even after two days of sleep recovery [15]. In rodents, the increased hippocampal BBB permeability remains impaired after 2 h of resting opportunity [16, 18]. A meta-analysis of several studies performed in sleep deprived mice indicated that 3 h of sleep recovery is not enough time to restore the basal hippocampal gene expression of cirpb and rbm3 [19], which are associated with altered expression of stress response and cell survival genes, suggesting that sleep loss has persistent effects in the hippocampus after short resting periods.

There are few studies determining the effects of resting in hippocampal protein expression and electrical activity. The aim of the present study is to determine the molecular, cellular and behavioral effects of 4 h sleeping opportunity following 10 days of SR induced by the modified multiple platform method (MMPM). With this aim in mind, we analyzed the molecular footprint of SR, with or without the recovery period, by using immunofluorescence analysis of neuroplasticity and neuroinflammation markers along with Western Blot analysis of memory-related kinase ERK phosphorylation levels, in the hippocampus. In addition, in a different set of animals, we assessed the effect of SR and the following recovery opportunity in hippocampal rhythms using LFP recordings. Finally, we assess how MMPM affects context and object recognition memory. We discuss the data in terms of how SR-induced molecular and cellular alterations could explain short-term and, possibly, long-term cognitive alterations.

## Materials and Methods

### Animals

This study was conducted in accordance with the guidelines and requirements of the World Medical Association Declaration of Helsinki (1964), approved by the ethics committee of the Faculty of Medicine, UNAM (protocol 032-2023 FM/0I/102/2023 Facultad de Medicina, UNAM), and regulated by the Mexican Official Norm NOM-062-ZOO-1999 to minimize animal suffering.

Male Wistar rats (280–350 g weight, 3 months old) were obtained from the *Unidad Académica Bioterio* (UAB) from the Faculty of Medicine, *Universidad Nacional Autónoma de México*. Animals were maintained under a 12:12 h light–dark cycle (lights were on from 8:00 = ZT 0 to 20:00 = ZT 12) with access to tap water and standard laboratory chow (5001 PMI Nutrition International Inc., Greenwood, MO) a*d libitum* throughout the experiments. Room temperature was maintained at 22 ± 2 °C with constant filtered air flow. All animals were habituated for 7 days prior to randomly assigning them into one of the four different experimental cohorts that will be described later.

### Sleep Restriction by the Modified Multiple Platform Method (MMPM)

To induce SR, we performed the modified multiple platform method (MMPM). The MMPM method prevents social isolation stress, as two rats from the same social group are placed together in both the restriction cage and their respective home cage. In addition, we previously showed that this SR protocol does not increase serum corticosterone levels, at least when measured on the tenth day, at the end of the last two hours of the resting phase [53], this procedure avoids immobilization stress since each restriction cage presents three platforms. and that is known to completely abolish REM sleep and to reduce NREM sleep by approximately 31% [18, 20].

**Fig. 1:**
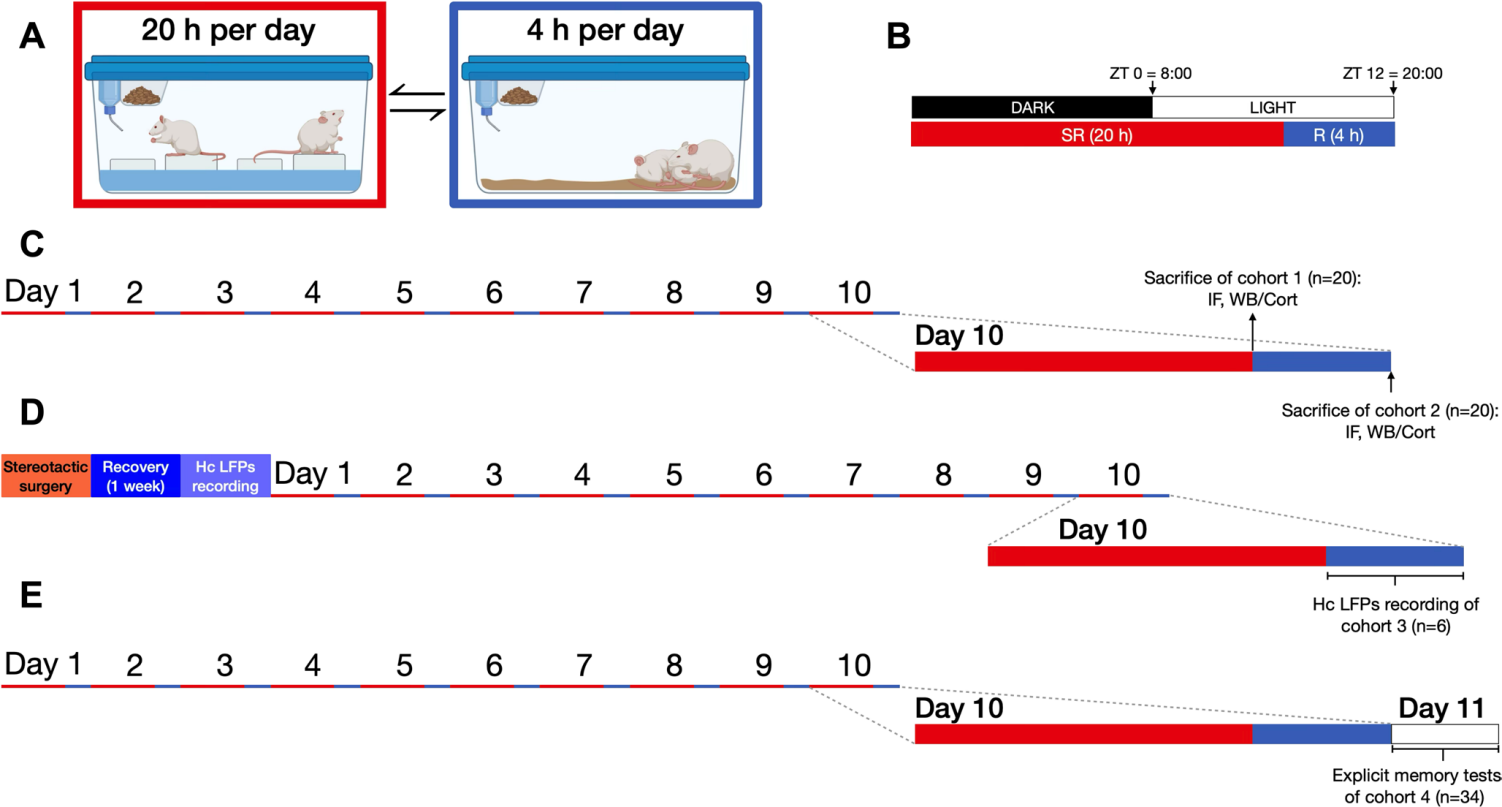
Experimental design of the modified multiple-platform method (MMPM) for sleep restriction. **A** Diagram of the MMPM protocol. Animals were placed in the SR cage (red border) for 20 h/day and then returned to their home cages for 4 h/day to allow recovery sleep (blue border). This schedule was repeated for 10 consecutive days. **B** Daily timeline aligned with the light–dark cycle. Black and white bars represent the dark and light phases, respectively. The SR period (red bar) was conducted from lights on (ZT0 = 08:00) to ZT8 = 16:00, continuing until ZT12 = 20:00, when animals were returned to their home cages for a 4 h recovery opportunity (blue bar) during the onset of the dark phase (ZT8–ZT12). **C** Experimental design for cohorts 1 and 2, designated for hippocampal protein analysis by Western blot (WB) and immunofluorescence (IF). Animals were sacrificed either immediately after SR (ZT8, cohort 1) or after a 4 h recovery period (ZT12, cohort 2). **D** Experimental design for cohort 3, used for hippocampal electrical activity analysis. Animals were implanted with hippocampal electrodes (orange bar) prior to the SR protocol, allowed one week for recovery (blue bar), and then local field potentials (LFPs) were recorded during the final 4 h recovery opportunity (ZT8–ZT12, cohort 3). **E** Experimental design for cohort 4, designated for behavioral testing. The novel object recognition test (NORT) and object location test (OLT) were performed on day 11, following completion of the SR protocol and a 4 h recovery opportunity (ZT8–ZT12, white bar)

To induce SR, rats (SR) were kept awake by placing them in pairs inside of acrylic cages (53 cm x 43 cm x 20 cm) filled with 3 cm of water and inside of which three platforms of 7 × 6 cm each (diameter and height, respectively) were placed in order to allow the rats to stand, sit and lie down; however whenever the animals lost muscle tone, they fell into the water, thus, waking them up. Rats remained in these restriction cages for 20 h (20:00–16:00 h) during 10 consecutive days and were allowed to rest in their home cages for the remaining 4 h (16:00–20:00 h) (Fig. 1A and B).

## Experimental Design

The first cohort included 20 rats, divided into a control group (CNT, n 10), which remained in their home cages throughout the protocol, and a sleep-restricted group (SR, *n* = 10). After the final 20 h of sleep restriction on day 10, SR animals were euthanized immediately upon removal from the restriction cage, along with time-matched CNT counterparts. Brains were collected either after perfusion for immunofluorescence (IF, n 4 per condition) or after decapitation for Western blot (WB, *n* = 6 per condition) (Fig. 1C). Trunk blood samples from decapitated animals were collected to determine corticosterone concentrations (blood could not be collected from 2 SR rats).

The second cohort initially included 20 rats (10 SR and 10 CNT). SR animals underwent the same sleep restriction protocol but, following the last 20 h of restriction on day 10, were allowed 4 h of recovery sleep in their home cages. CNT rats remained in their home cages for the entire protocol. At the end of the 4 h recovery period, both groups were sacrificed simultaneously. Brains were collected either after perfusion for IF (n 4 per condition) or after decapitation for WB (*n* = 6 per condition; one SR rat died during the protocol, and one CNT sample degraded) (Fig. 1C). Trunk blood was also obtained from decapitated animals to determine corticosterone concentrations (two CNT samples could not be obtained). Paired time-matched controls were included in this cohort because the hippocampus exhibits circadian oscillations in molecular and cellular markers [21–26]. In our design, animals were sacrificed either immediately after sleep restriction (ZT8, light phase) or after a 4 h recovery period coinciding with the onset of the dark phase (ZT12, active phase). Including time-matched controls ensured that the effects of sleep restriction could be distinguished from circadian influences.

A third cohort consisted of 6 rats implanted with hippocampal electrodes in CA1 to record local field potentials (LFPs) before (non-restricted baseline) and after the SR protocol (Fig. 1D). The baseline recording was obtained on day 7 after surgery, between 16:00 and 20:00 h, corresponding to the 4 h resting period later used in the SR protocol. The following day, all animals began the MMPM protocol for 10 consecutive days. A second recording was obtained during the 4 h recovery opportunity at the end of the restriction protocol. In this design, each animal served as its own control, allowing paired comparisons of hippocampal activity before and after SR. This approach also minimized variability and avoided the technical complications of maintaining electrodes in paired animals housed under water-exposure conditions during the restriction phase.

Finally, a fourth cohort consisted of 34 rats used for explicit memory testing: 20 for the NORT (CNT n 10, SR n 10; one SR rat was excluded because it jumped out of the testing box) and 14 for the OLT (CNT *n* = 8, SR n 6; one CNT and one SR were excluded because their video recording were incomplete).

### Immunofluorescence

Animals were euthanized either immediately after the SR protocol or following 4 h of recovery sleep by intraperitoneal injection of an overdose of sodium pentobarbital (Aranda, 200 mg/kg). Once deeply anesthetized, and just before cardiac arrest, animals underwent transcardial perfusion with 0.9% saline solution for 5 min (15 mL/min), followed by 4% ice-cold paraformaldehyde (PFA) in phosphate-buffered saline (PBS) for 5 min.

Brains were collected, post-fixed in 4% ice-cold PFA for 48 h at room temperature under continuous gentle agitation, and subsequently cryoprotected in 30% sucrose/0.05% sodium azide in PBS at 4 °C until use. Serial coronal Sect. (30 μm thick) of the hippocampus (Hc; Bregma − 2.52 mm to − 3.24 mm, Paxinos & Watson, 2007) were obtained with a Leica CM1850 UV cryostat, collected in cryoprotectant solution, and stored at 4 °C until immunofluorescence (IF) processing.

For each animal, four sections containing the bilateral dorsal Hc were pre-incubated for 10 min in 1% H₂O₂ to block endogenous peroxidase activity. After a PBS wash, sections were incubated overnight at room temperature under constant agitation with the following primary antibodies: IBA-1 (Abcam, ab5076, 1:1000) and Synaptophysin (Syn; Santa Cruz Biotechnology, sc-17750, 1:2000).

The next day, sections were washed with PBS and incubated for 2 h with secondary antibodies: CY3-conjugated donkey anti-goat (Jackson ImmunoResearch, 705-165-147, 1:500) and Alexa Fluor 488-conjugated donkey anti-mouse (Jackson ImmunoResearch, 715-545-150, 1:500). Sections were then washed with PBS and mounted on gelatin-coated slides. After drying, slides were incubated for 10 min with DAPI solution (Invitrogen, 4′,6-diamidino-2-phenylindole, dihydrochloride, D1306, 1 µg/mL) and cover-slipped using anti-fade fluorescence mounting medium (Abcam, ab104135).

### Syn and IBA-1 Immunoreactivity and Morphological Analyses

Confocal micrographs (20×) of the hippocampal CA1 and CA3 regions (3–4 fields per animal) were acquired using a ZEISS LSM 880 confocal microscope. Quantitative analysis of optical density (OD) per section was performed in FIJI software [27]. Since the images exclusively contained the regions of interest, no additional ROI tracing was required; OD was measured across the entire image. Data are presented as OD percentage normalized to controls for both Synaptophysin (Syn) and IBA-1 immunofluorescent staining.

To determine the number of IBA-1–positive cells, 3–4 micrographs from CA1 and CA3 regions were analyzed per animal. Cells were counted based on the following criteria: (1) only complete cells were included, and (2) both DAPI and IBA-1 signals were required.

For morphological analysis of IBA-1–positive cells, 3–4 micrographs per region per animal were evaluated. A 10 × 10 grid was applied in FIJI, and 3 random cells per region were selected using the Excel randomization function. Each selected cell was cropped and processed with a standardized script to generate binary and skeletonized images. Images were converted to 8-bit grayscale, filtered to improve contrast and remove background, and then transformed into binary and skeletonized formats. This processing was performed independently by two researchers blinded to the experimental conditions.

Sholl analysis was carried out on binary images with the FIJI Neuroanatomy plugin to characterize glial arborization. Concentric circles (5 μm apart) were drawn from the soma, and intersections of processes with the shells were quantified [28]. Branch number was assessed on skeletonized images using the FIJI Skeleton plugin.

### Western Blot

Either immediately after sleep restriction or following 4 h of recovery sleep in the MMPM protocol, animals were euthanized by decapitation. Hippocampi from CNT and SR groups were collected and stored at − 80 °C until processing. Tissue was homogenized in NP-40 lysis buffer supplemented with protease and phosphatase inhibitors, and samples were centrifuged at 13,500 rpm for 10 min at 4 °C. The supernatant was collected, and protein concentration was determined by spectrophotometry at 260 nm (Epoch BioTek).

For immunoblotting, 50 µg of protein were resolved on 10% SDS-PAGE gels, transferred to polyvinylidene fluoride (PVDF) membranes, and blocked with 5% (w/v) nonfat milk in PBS-Tween 20 for 45 min. Membranes were incubated overnight at 4 °C with the following primary antibodies: anti-Synaptophysin (Syn; Santa Cruz Biotechnology, sc-17750, 1:1000), anti-ERK (Cell Signaling, cat. 4695, 1:2000), anti-phospho-ERK (Santa Cruz Biotechnology, sc-101760, 1:400), and anti-β-actin (Santa Cruz Biotechnology, sc-69879, 1:1000).

The next day, membranes were incubated with biotinylated secondary antibodies donkey anti-mouse (Jackson ImmunoResearch, 715-065-150, 1:2000) or donkey anti-rabbit (Jackson ImmunoResearch, 711-065-152, 1:2000), followed by avidin–biotin complex amplification (Vectastain Elite ABC-HRP Kit, PK-6100, 2.5:1000). Signals were visualized using a chemiluminescence detection system (Bio-Rad, 1705060).

Semi-quantitative analysis was performed using the Gel Analyzer tool in FIJI. β-actin optical density was used as the loading control for normalization. Protein expression was calculated as the ratio of the target protein to β-actin, and fold changes were determined relative to the CNT group (set to 1). Original PVDF membrane images used for analysis are provided in Supplementary Figs. 1–4.

### Hippocampal Local Field Potentials

Local field potentials (LFPs) provide a reliable measure of brain electrical activity [29, 30]. To assess the effects of SR on hippocampal function, six male rats were implanted with a recording electrode during stereotaxic surgery. Animals were anesthetized with a ketamine/xylazine mixture (90/10 mg/kg, i.p.) and positioned in a stereotaxic frame. After exposing Bregma, a stainless-steel recording electrode was implanted in the left dorsal CA1 region of the hippocampus at the following coordinates: AP = − 3.8 mm; L = 2.0 mm; V = 3.0 mm. The representative electrode location is shown in Supplementary Figure S5.

Two stainless-steel screws were anchored to the skull: one above the dura mater of the right motor cortex (indifferent monopolar ground) and another on the left occipital bone (fixation). Electrodes were connected to a 5-pin micro-USB connector and secured with dental acrylic. The wound was treated with topical gentamicin (0.3%) and sutured. Animals were housed individually in acrylic cages during recovery. For the first three postoperative days, rats received enrofloxacin (5 mg/kg, i.p., every 24 h).

Two LFP recordings were obtained from the same animal. The first was performed on day 7 after surgery, between 16:00 and 20:00 h, serving as a baseline (BL). This time window corresponded to the 4 h resting period later used in the SR protocol. The following day, all six rats began the MMPM protocol for 10 consecutive days. The second recording was obtained during the 4 h recovery opportunity at the end of the SR protocol. In this way, each animal served as its own control, minimizing variability and avoiding the technical challenges of maintaining electrodes in paired animals housed under MMPM conditions.

For LFP recordings, rats were placed in acrylic boxes (30 × 22 × 22 cm) and connected to a video-LFP acquisition system (BE Light, EB Neuro S.p.A., Florence, Italy) via flexible cables that allowed free movement. Data were sampled at 256 Hz, low-pass filtered at 0.3 Hz, and high-pass filtered at 70 Hz using Galileo NT software (EB Neuro, Italy). Relative power across frequency bands was calculated using automated Fast Fourier Transform analysis.

### Novel Object Recognition and Object Location Test

The novel object recognition test (NORT) and the object location test (OLT) are widely used paradigms to assess explicit memory [25, 26]. We employed both tests to evaluate the effects of SR on this type of memory.

For the NORT, 20 rats were randomly assigned to two groups: control animals (CNT, *n* = 10), which remained in their home cages for 10 days, and sleep-restricted animals (SR, *n* = 10; one animal was excluded after escaping from the testing arena during trials). For the OLT, 14 rats were used (CNT, *n* = 8; SR, *n* = 6); one animal from each group was excluded due to incomplete video recordings.

After the final restriction period, SR animals were returned to their home cages for a 4 h resting opportunity. Thereafter, either the NORT or the OLT was performed at 20:00 h, corresponding to the beginning of the dark/activity phase. Tests were not conducted immediately after SR, as pilot experiments indicated that animals tended to fall asleep rather than explore the objects (data not shown).

Behavioral testing was conducted in a 45 × 45 × 45 cm open wooden box (without lid) and consisted of three phases: habituation, training, and test. To minimize disruption of the light/dark cycle, all sessions were conducted under red light. The habituation and training phases were identical for both NORT and OLT:

1. Habituation: Rats were transferred to the behavioral testing room in their own home cages and left undisturbed for 1 h to acclimate. Next, each rat was placed in one of two parallel empty arenas for habituation (one CNT and one SR animal simultaneously) for 5 min before being returned to its home cage.
2. Training: One hour later, two identical objects (either an upside-down 100 mL glass beaker or a 60 mL Coplin staining jar; objects A and A′ respectively) were placed at diagonally opposite corners of the testing box. Animals were allowed to explore the objects for 5 min before being returned to their home cages.
3. NORT trial: One hour after training, animals were placed in the testing box with one familiar object (A) and one novel object (B, a glass object of similar size but different shape from the familiar object). Both objects were positioned in the same locations as during training. Exploration was allowed for 5 min.
4. OLT trial: One hour after training, animals were placed in the testing box with the same two identical objects used during training. One object (A) remained in its original location, while the other (A′) was displaced to a new location. Exploration lasted 5 min.

All trials were video-recorded for later analysis. Exploration times were scored by two independent researchers blind to the experimental conditions. The percentage of investigation time during training was calculated as TA′/(TA′+TA)×100, where TA′ is the exploration time of object A′ and TA is the exploration time of object A. For the test trials, the percentage of investigation time was calculated as TB/(TB + TA)×100, where TB is the exploration time of the novel (NORT) or relocated (OLT) object, and TA is the exploration time of the familiar (NORT) or non-relocated (OLT) object.

### Corticosterone Radioimmunoassay

Blood samples from euthanized animals were collected in 2.5 mL microtubes and centrifuged at 11,700 rpm for 15 min. Plasma was aliquoted (60 µL) and stored at − 80 °C until analysis. Corticosterone concentrations were determined using the Corticosterone Double Antibody RIA Kit (MP Biomedicals, cat. 07120102), following the manufacturer’s instructions. Briefly, plasma and standard curve samples were diluted 1:400 with RIA buffer (50 mM PBS containing 0.1% gelatin). Corticosterone-I^125 tracer and anti-Corticosterone antibody were then added, and samples were incubated for 16 h at 4 °C. Subsequently, precipitant solution and an equal volume of polyethylene glycol were added to each sample, which was centrifuged at 4000 rpm for 15 min at 4 °C. The supernatant was discarded, and radioactivity in the pellet was quantified using a Genesys (LTI Laboratory Technologies) γ-counter. All samples were analyzed in duplicate, and blood corticosterone concentrations are expressed in ng/µL.

### Statistical Analysis

Normality of the data distribution was assessed using the Shapiro–Wilk test. Parametric analyses were applied when data met the assumption of normality. Behavioral tests, hippocampal IBA-1 optical density (OD), and the number of IBA-1 + cells were analyzed by unpaired Student’s t-test. The number of branches immediately after restriction was analyzed by unpaired Student’s t-test, while the number of intersections was evaluated by two-way ANOVA followed by Sidak’s post hoc test. Parametric data are presented as mean ± standard error (SEM).

When data did not meet the assumption of normality, non-parametric tests were used. Specifically, the number of branches after 4 h of rest was analyzed by the Mann–Whitney U test. Hippocampal LFPs were analyzed by Student’s t-test, except for total spectral power, which was analyzed using the Wilcoxon signed-rank test. Non-parametric data are presented as median with range.

All statistical analyses were performed with Prism 10 (GraphPad Software), and statistical significance was set at *p* < 0.05. Grubb’s test with alpha = 0.05 was used to de-termine outliers that were excluded.

## Results

### Ten Days of Sleep Restriction Induce Region-Specific Changes in Hippocampal IBA-1 Immunoreactivity and Microglial Morphology

Since different sleep deprivation and sleep restriction protocols induce changes in the expression of several macrophage/microglial markers [31, 32], we evaluated hippocampal IBA-1 immunoreactivity (IR) immediately after the MMPM protocol and after 4 h of recovery sleep.

**Fig. 2:**
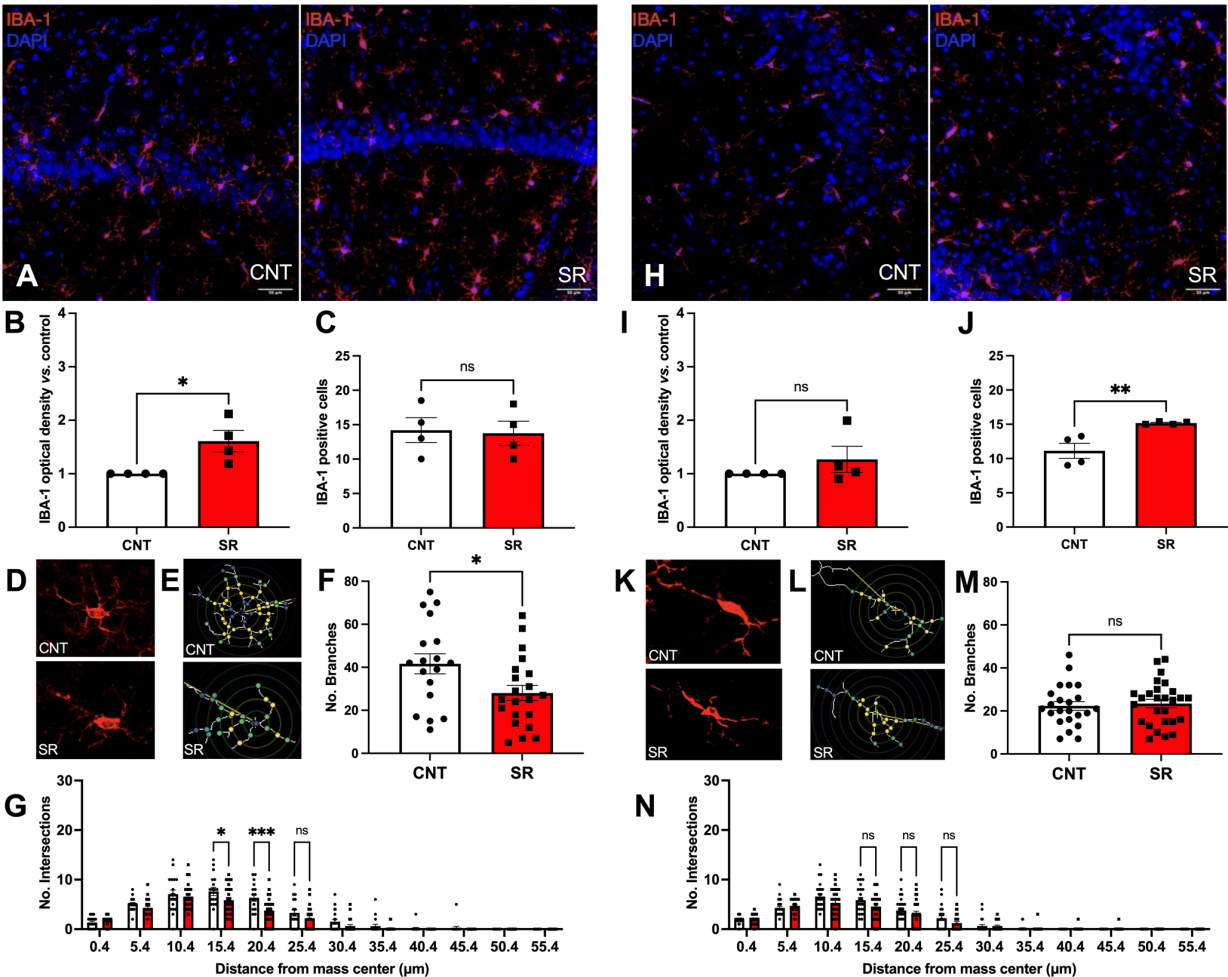
Ten days of sleep restriction alters hippocampal IBA-1 positive cells immediately after the MMPM protocol. **A** Representative images of IBA-1 positive cells (red) and cell nuclei (DAPI, blue) in the CA1 region of control (CNT) and sleep-restricted (SR) animals. Scale bar: 50 μm. **B** Optical density (OD) of IBA-1 immunoreactivity in CA1 for CNT (white bars) and SR (red bars) animals. **C** Number of IBA-1 + DAPI positive cells in CA1. **D** Representative images of IBA-1 positive cells from CNT and SR animals. **E** Binary and skeletonized versions of the images in D. Concentric circles (5 μm apart) were plotted around the cell center of mass, with colored circles indicating branch intersections. **F** Number of branches per CA1 IBA-1 positive cell. **G** Number of intersections per CA1 IBA-1 positive cell. **H** Representative images of IBA-1 positive cells (red) and nuclei (blue) in CA3. Scale bar: 50 μm. **I** Optical density of IBA-1 immunoreactivity in CA3. **J** Number of IBA-1 + DAPI positive cells in CA3. **K** Representative images of IBA-1 positive cells in CNT and SR animals. **L** Binary and skeletonized versions of the images in K, with concentric circles (5 μm apart) plotted around the cell center of mass; colored circles indicate branch intersections. **M** Number of branches per CA3 IBA-1 positive cell. **N** Number of intersections per CA3 IBA-1 positive cell. Results are expressed as mean ± SEM, except for the number of branches, which is shown as median with range. Statistical analysis: optical density, IBA-1 positive cells, and intersections were analyzed with unpaired Student’s t-tests; the number of branches was analyzed with the Mann–Whitney test. Two-way ANOVA was used for Sholl intersection analysis. Asterisks represent statistical significance: *p < 0.05, **p < 0.01, ***p < 0.001

In the CA1 region (Fig. 2A), SR animals showed a significant increase in IBA-1 IR immediately after restriction compared with controls (Student’s t-test, t = 3.046, df = 6, *p* = 0.0226; Fig. 2B). In contrast, no differences were observed in CA3 IR (Student’s t-test, t = 1.087, df = 6, *p* = 0.3186; Fig. 2I). Notably, the increase in CA1 IBA-1 IR was not associated with changes in the number of IBA-1 + cells (Student’s t-test, t = 0.1823, df = 6, *p* = 0.4307; Fig. 2C). Conversely, the CA3 region displayed a significant increase in IBA-1 + cell number (Student’s t-test, t = 3.698, df = 6, *p* = 0.0051; Fig. 2J), despite no changes in IR between CNT and SR animals.

Skeleton analysis revealed a reduction in branch number in CA1 of SR rats (Mann–Whitney U = 111.5, *p* = 0.0142; Fig. 2F). In addition, Sholl analysis in CA1 showed that SR IBA-1 + cells had significantly fewer intersections than those of the CNT group (Two-way ANOVA, F [1, 40] = 4.706, *p* = 0.0360; Fig. 2G), with differences at 15.4 μm (*p* = 0.0271) and 20.4 μm (*p* = 0.0001), indicating that 10 days of SR induced morphological changes consistent with shorter and less ramified processes in CA1 microglia/macrophages.

In contrast, CA3 IBA-1 + cells showed no changes in either branch number (Mann–Whitney U = 281.5, *p* = 0.2891; Fig. 2M) or number of intersections (Two-way ANOVA, F [1, 49] = 1.579, *p* = 0.2148; Fig. 2N) after SR. Taken together, our CA1 and CA3 IBA-1 IR and morphological analyses demonstrate that SR alters hippocampal IBA-1 expression and microglial morphology in a region-specific manner.

### Hippocampal IBA-1 Immunoreactivity is Partially Restored After 4 h of Resting Opportunity, While Microglial Morphology Remains Altered

**Fig. 3:**
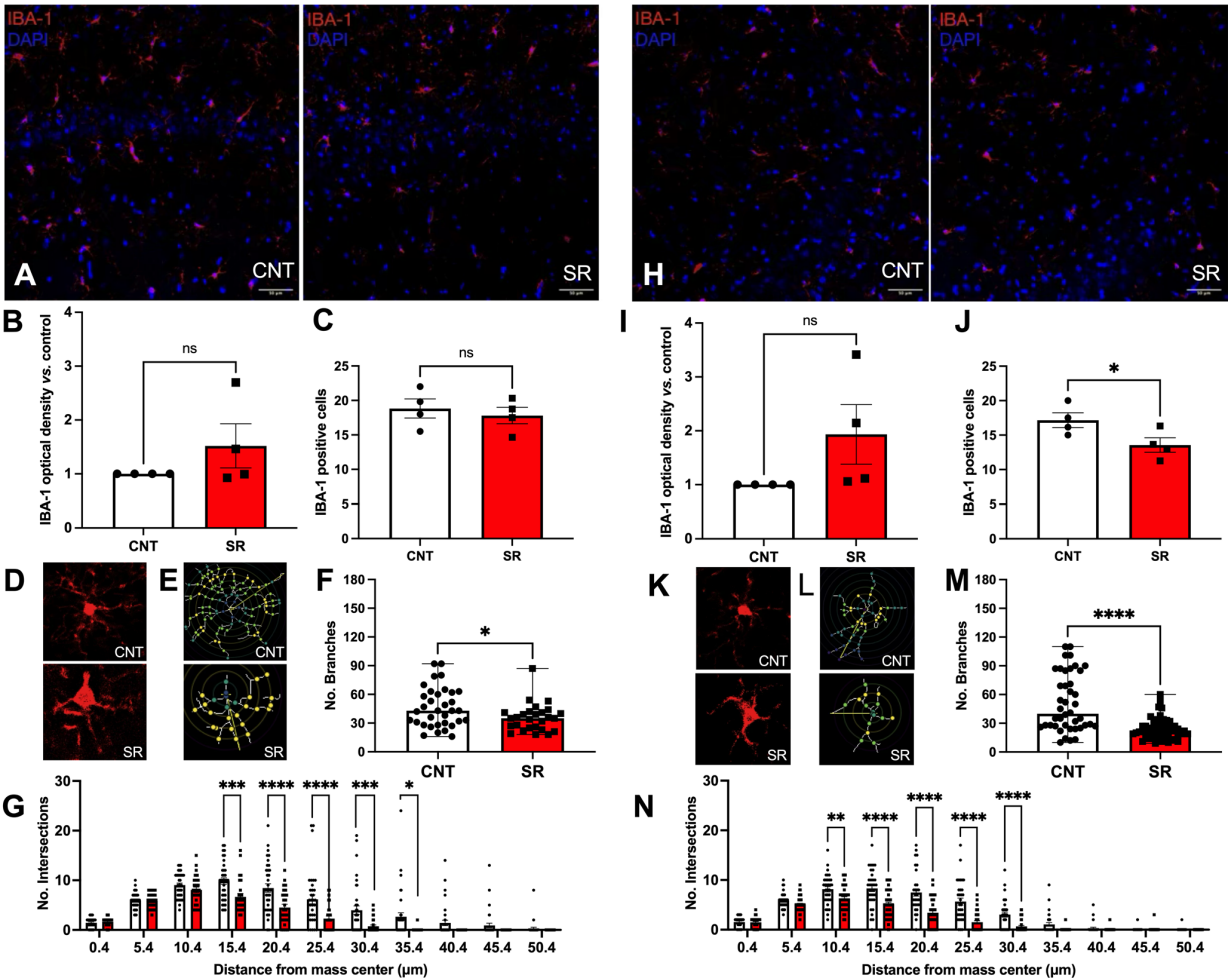
Alterations in hippocampal IBA-1 positive cells persist after 4 h of rest following 10 days of sleep restriction. **A** Representative images of IBA-1 positive cells (red) and nuclei (DAPI, blue) in CA1 of CNT and SR animals. Scale bar: 50 μm. **B** Optical density of IBA-1 immunoreactivity in CA1. **C** Number of IBA-1 + DAPI positive cells in CA1. **D** Representative IBA-1 positive cells from CNT and SR rats. **E** Binary and skeletonized versions of D. Concentric circles (5 μm apart) plotted around the cell center of mass; colored circles indicate branch intersections. **F** Number of branches in CA1 IBA-1 positive cells. **G** Sholl analysis showing number of intersections in CA1. (H) Representative images of IBA-1 positive cells (red) and nuclei (blue) in CA3. Scale bar: 50 μm. **I** Optical density of IBA-1 immunoreactivity in CA3. **J** Number of IBA-1 + DAPI positive cells in CA3. **K** Representative IBA-1 positive cells from CNT and SR rats. **L** Binary and skeletonized versions of K, with concentric circles (5 μm apart); colored circles indicate branch intersections. **M** Number of branches in CA3 IBA-1 positive cells. **N** Sholl analysis showing number of intersections in CA3. Results are expressed as mean ± SEM, except for branch number (median with range). Statistical analysis: optical density, IBA-1 positive cells, and intersections were analyzed with unpaired Student’s t-tests; branch number with the Mann–Whitney test; intersections with two-way ANOVA. Asterisks indicate statistical significance: *p < 0.05, **p < 0.01, ***p < 0.001, ****p < 0.0001

Next, we evaluated hippocampal IBA-1 IR and morphology after 4 h of recovery sleep. In CA1, no differences were found between CNT and SR groups in IBA-1 IR (Student’s t-test, t = 1.269, df = 6, *p* = 0.1258; Fig. 3B) or in the number of IBA-1 + cells (Student’s t-test, t = 0.5595, df = 6, *p* = 0.2980; Fig. 3C). Similarly, CA3 IBA-1 IR did not differ between groups (Student’s t-test, t = 1.689, df = 6, *p* = 0.0711; Fig. 3I). However, in contrast to the increase observed immediately after SR, the number of IBA-1 + cells in CA3 was lower in SR animals compared to controls after 4 h of rest (Student’s t-test, t = 2.395, df = 6, *p* = 0.0268; Fig. 3J).

Morphological analyses revealed persistent alterations despite the resting period. Skeleton analysis showed that CA1 IBA-1 + cells in SR rats still had fewer branches compared to controls (Mann–Whitney U = 320.5, *p* = 0.0220; Fig. 3F). Likewise, CA3 IBA-1 + cells also exhibited a significant reduction in branch number after SR (Mann–Whitney U = 461.5, *p* < 0.0001; Fig. 3M). Sholl analysis further confirmed these findings. In CA1, SR IBA-1 + cells had significantly fewer intersections than controls after 4 h of rest (Two-way ANOVA, F [1, 61] = 11.29, *p* = 0.0014; Fig. 3G). Similarly, CA3 IBA-1 + cells showed a pronounced reduction in intersections compared to controls (Two-way ANOVA, F [1, 73] = 29.28, *p* < 0.0001; Fig. 3N).

Together, these results indicate that although IBA-1 IR in the hippocampus shows partial recovery after a short resting opportunity, microglial morphology remains altered, suggesting that 4 h of sleep is insufficient to normalize the structural changes induced by subchronic SR.

### Hippocampal Synaptophysin Immunoreactivity is Down-Regulated Even After 4 h of Resting Opportunity

Changes in hippocampal IBA-1 IR have been associated with alterations in microglial function. Previous studies have shown that after SR, CNS microglia increase the phagocytosis of synaptic proteins [17]. To assess whether the changes in IBA-1 expression observed here could be linked to alterations in synaptic density, we next analyzed the expression of the presynaptic vesicle protein synaptophysin (Syn).

**Fig. 4:**
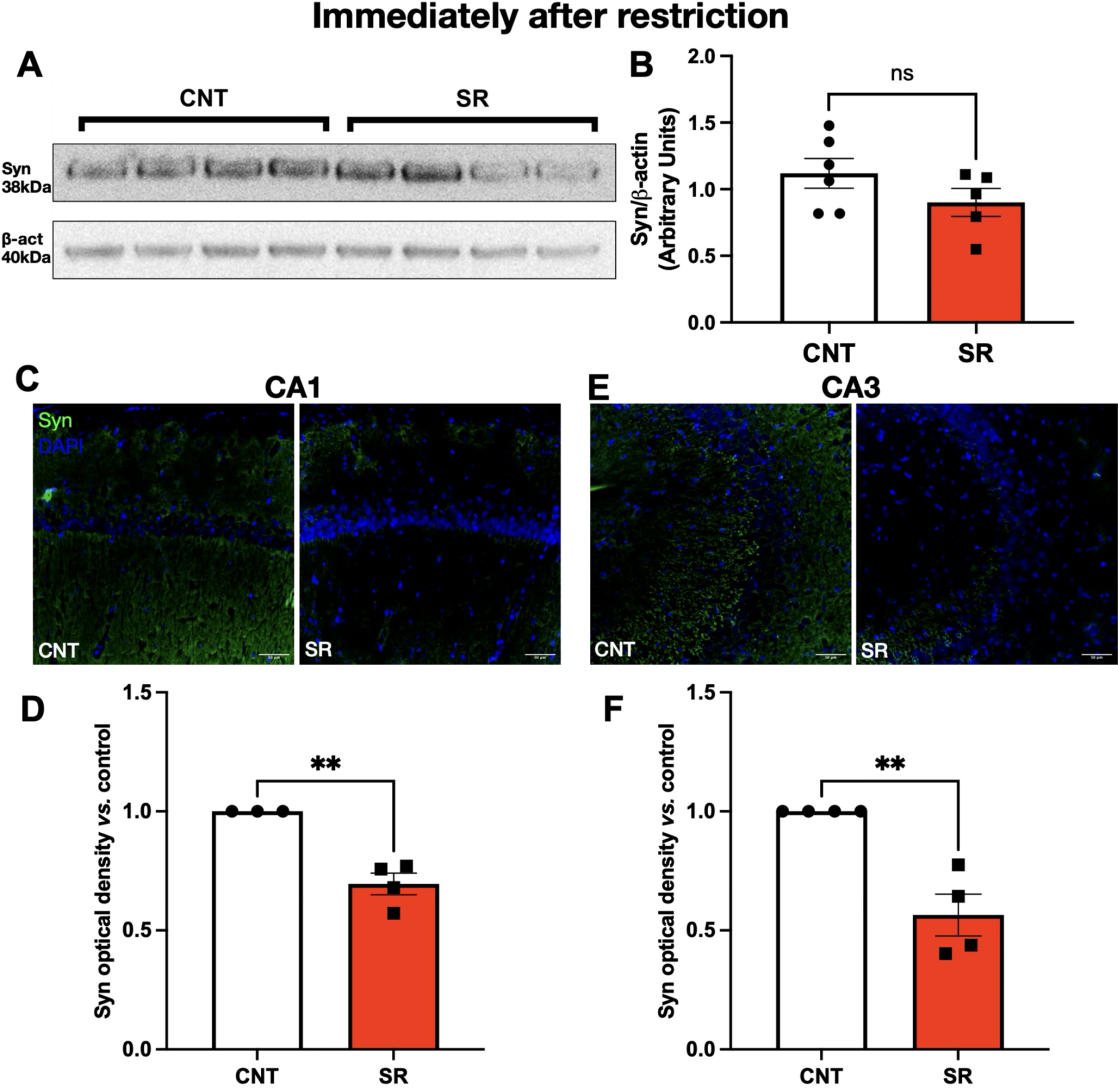
Ten days of sleep restriction decreases hippocampal synaptophysin levels immediately after the MMPM protocol. **A** Representative Western blot of synaptophysin (Syn, 38 kDa) and β-actin (40 kDa) from control (CNT) and sleep-restricted (SR) animals. **B** Quantification of Syn/β-actin optical density ratio in CNT (white bars) and SR (red bars). **C** Representative immunofluorescence images of Syn (green) and nuclei (DAPI, blue) in the CA1 region of CNT and SR rats. Scale bar: 50 μm. **D** Quantification of Syn optical density in CA1. **E** Representative immunofluorescence images of Syn (green) and nuclei (blue) in the CA3 region. Scale bar: 50 μm. **F** Quantification of Syn optical density in CA3. Results are expressed as mean ± SEM. Statistical analysis was performed with unpaired Student’s t-tests. Asterisks indicate statistical significance: **p < 0.01

**Fig. 5:**
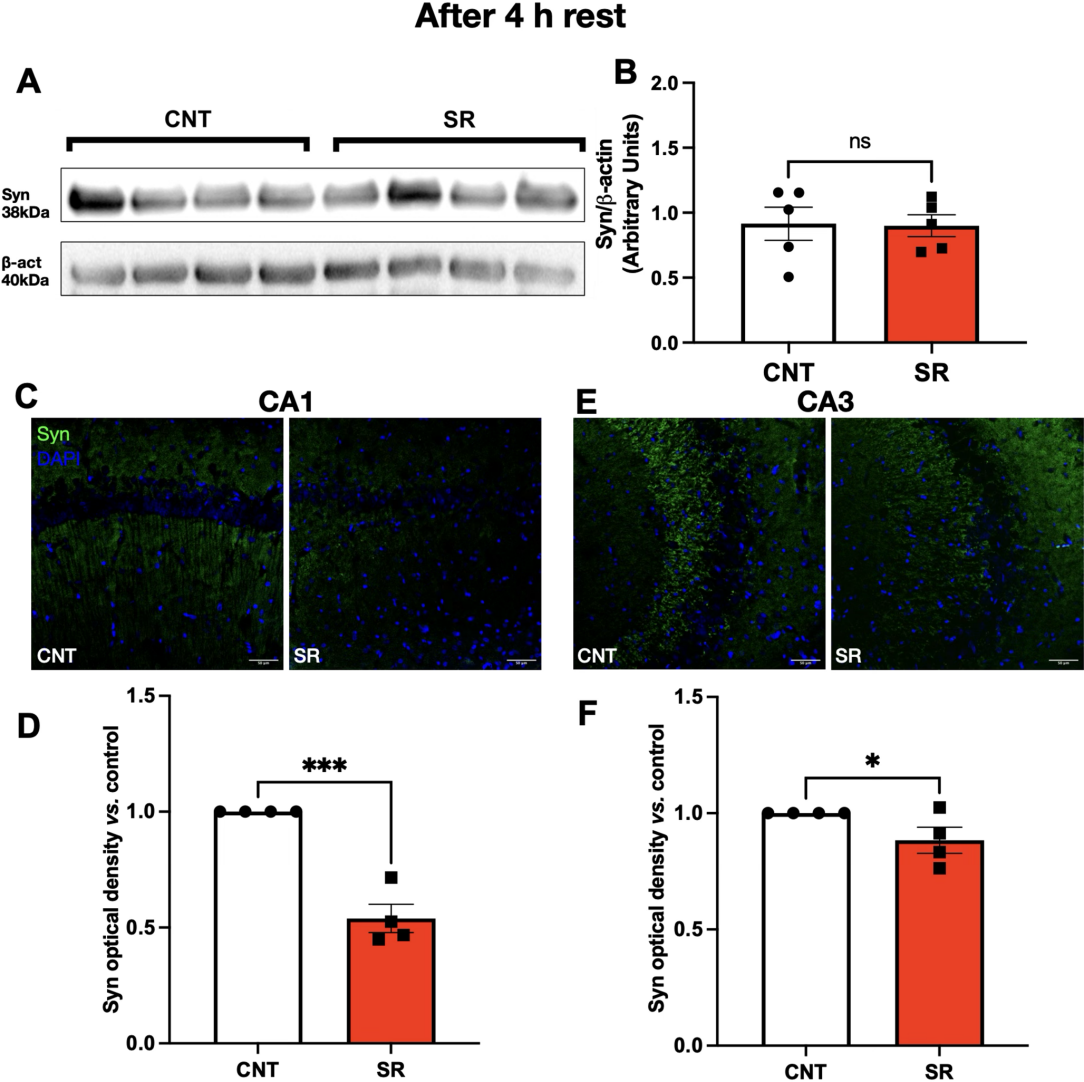
Decrease in hippocampal synaptophysin levels induced by 10 days of sleep restriction persists after 4 h of rest. **A** Representative Western blot of synaptophysin (Syn, 38 kDa) and β-actin (40 kDa) from control (CNT) and sleep-restricted (SR) animals. **B** Quantification of Syn/β-actin optical density ratio in CNT (white bars) and SR (red bars). **C** Representative immunofluorescence images of Syn (green) and nuclei (DAPI, blue) in the CA1 region of CNT and SR rats. Scale bar: 50 μm. **D** Quantification of Syn optical density in CA1. **E** Representative immunofluorescence images of Syn (green) and nuclei (blue) in the CA3 region. Scale bar: 50 μm. **F** Quantification of Syn optical density in CA3. Results are expressed as mean ± SEM. Statistical analysis was performed with unpaired Student’s t-tests. Asterisks indicate statistical significance: *p < 0.05, ***p < 0.001

Western blot analysis of hippocampal homogenates revealed no significant differences in Syn expression between CNT and SR animals, either immediately after restriction (Student’s t-test, t = 1.402, df = 9, *p* = 0.1946; Fig. 4B) or after 4 h of recovery sleep (Student’s t-test, t = 0.1018, df = 8, *p* = 0.9215; Fig. 5B).

Because homogenates represent the entire hippocampus, we next evaluated Syn expression specifically in CA1 and CA3 using immunofluorescence (IF). Immediately after SR, Syn IR was significantly decreased in SR rats compared to controls in both CA1 (Student’s t-test, t = 5.671, df = 5, *p* = 0.0024; Fig. 4D) and CA3 (Student’s t-test, t = 4.950, df = 6, *p* = 0.0026; Fig. 4F).

Importantly, Syn IR remained reduced even after 4 h of rest. SR rats continued to show significantly lower Syn levels in CA1 (Student’s t-test, t = 7.563, df = 6, *p* = 0.0001; Fig. 5D) and CA3 (Student’s t-test, t = 2.068, df = 6, *p* = 0.0420; Fig. 5F) compared to controls.

Together, these findings indicate that SR leads to a persistent reduction in hippocampal Syn expression that is not reversed by a short recovery period, consistent with disrupted presynaptic integrity.

### ERK Phosphorylation Increased After the Last 4 h of Resting

Since Syn expression was reduced after the SR protocol and remained decreased after a short resting opportunity, we next evaluated whether extracellular signal-regulated kinase (ERK) expression and phosphorylation (activation) were affected by SR. ERK has previously been shown to enhance hippocampal synaptic plasticity and promote long-term potentiation induction, while increased ERK phosphorylation correlates with higher synaptophysin immunoreactivity, reflecting enhanced synapse formation and/or maturation [33, 34]. Based on this evidence and our Syn results, we hypothesized that a reduction in the pERK/ERK ratio would be observed in the hippocampus after SR and would persist after the resting opportunity.

**Fig. 6:**
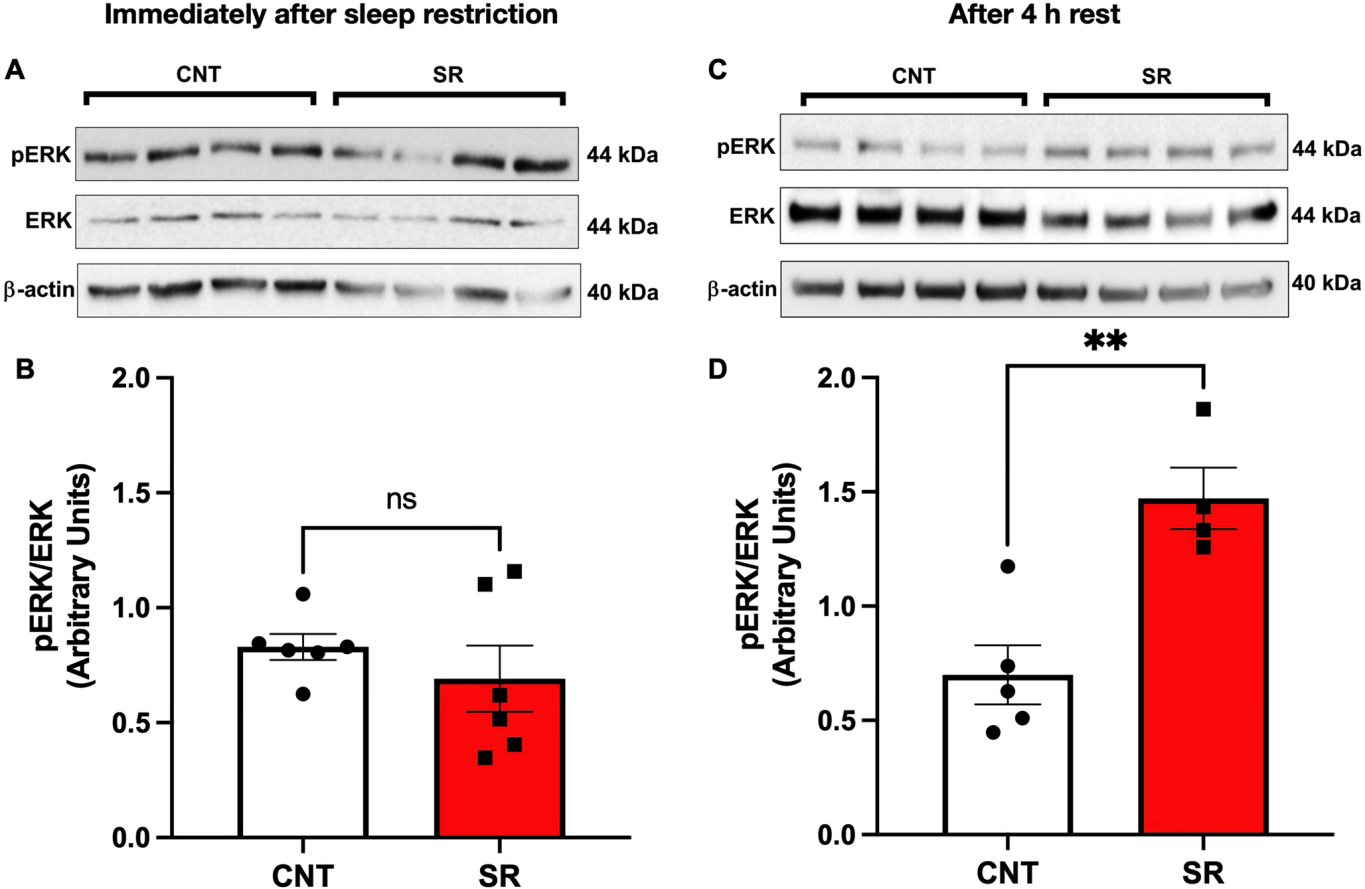
Sleep restriction increases the hippocampal pERK/ERK ratio after 4 h of rest. **A** Representative Western blot of phosphorylated ERK (pERK, 42/44 kDa), total ERK (42/44 kDa), and β-actin (40 kDa) from control (CNT) and sleep-restricted (SR) animals immediately after the SR protocol. **B** Quantification of pERK/ERK optical density ratio in CNT (white bars) and SR (red bars) immediately after SR. **C** Representative Western blot of pERK, total ERK, and β-actin from CNT and SR animals after 4 h of recovery. **D** Quantification of pERK/ERK optical density ratio in CNT and SR after 4 h of rest. Results are expressed as mean ± SEM. Statistical analysis was performed with unpaired Student’s t-tests. Asterisks indicate statistical significance: **p < 0.01

Contrary to this hypothesis, the hippocampus of SR rats showed no significant change in the pERK/ERK ratio immediately after the restriction compared with CNT animals (Student’s t-test, t = 0.8952, df = 10, *p* = 0.3917; Fig. 6B). Furthermore, after 4 h of rest, the SR group exhibited a significant increase in the pERK/ERK ratio relative to controls (Student’s t-test, t = 4.105, df = 7, *p* = 0.0045; Fig. 6D). These data indicate that recovery sleep induces a rebound—possibly compensatory—ERK activation in the hippocampus of SR animals.

### Hippocampal Electrical Activity is Impaired During the 4 h of Rest Following 10 Days of SR

Since the hippocampal pERK/ERK ratio increased while Syn IR and IBA-1 morphology remained altered after 4 h of rest, we hypothesized that hippocampal electrical activity during this recovery period would also be impaired. To test this, we implanted a recording electrode in CA1, the final relay of the trisynaptic pathway, where LTP is promoted during memory consolidation [35, 36].

**Fig. 7:**
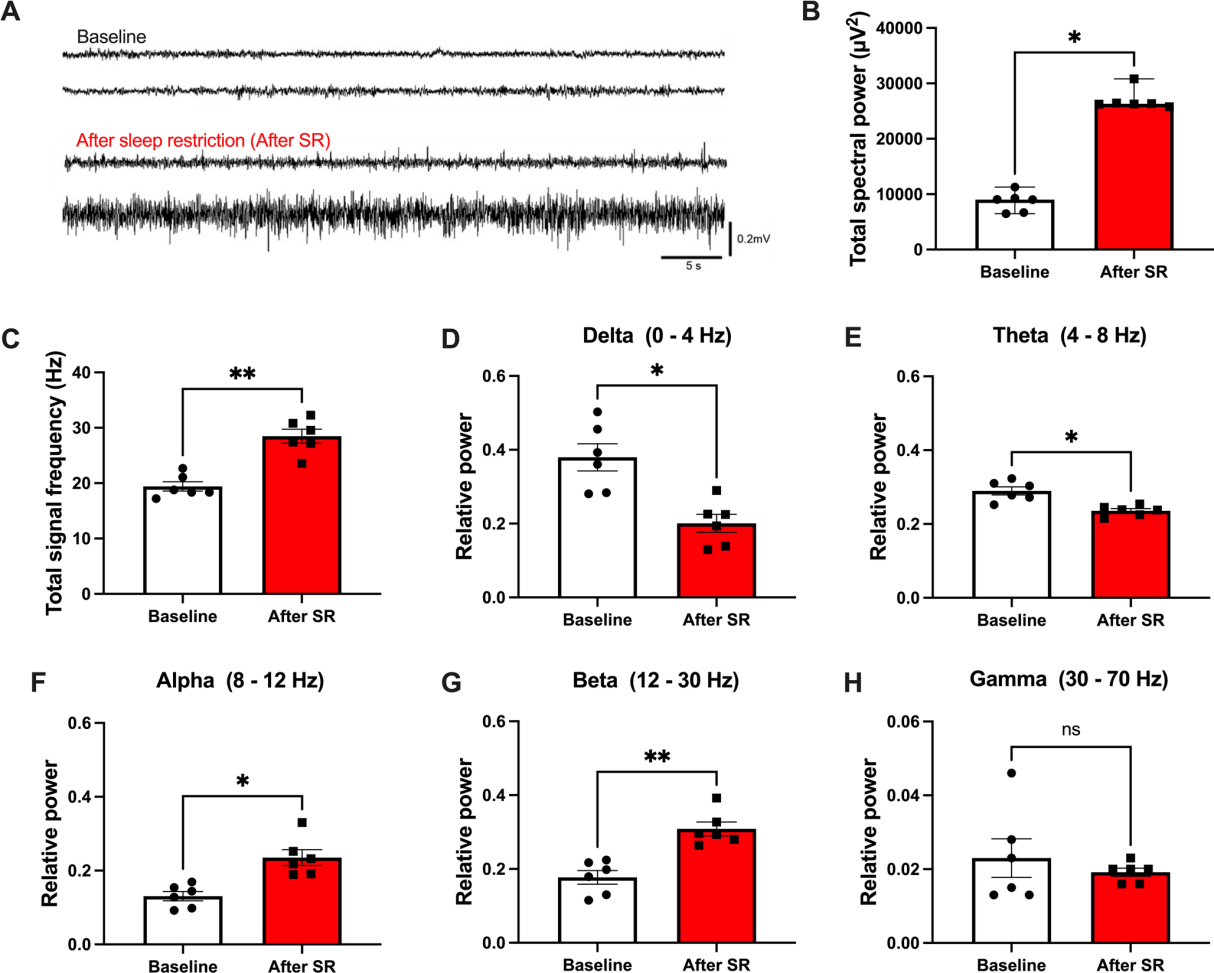
Sleep restriction alters hippocampal electrical activity during the 4 h recovery period. **A** Representative local field potential (LFP) traces of the hippocampus at baseline (before the 10-day SR protocol) and during the 4 h recovery period after SR. **B** Total spectral power. **C** Total signal frequency for baseline (white bars) and after SR (red bars). **D**–**H** Relative power of delta, theta, alpha, beta, and gamma waves recorded during the 4 h recovery period. Results are expressed as mean ± SEM for six animals, except for total spectral power, which is expressed as median with range. LFP recordings after SR were compared with baseline recordings from the same animals. Statistical analysis was performed with two-way ANOVA followed by Sidak’s post hoc test, except for total spectral power, which was analyzed with the Wilcoxon test. Asterisks indicate statistical significance: *p < 0.05, **p < 0.01

CA1 total spectral power was significantly increased in SR animals compared to controls (Wilcoxon test, W = 21, *p* = 0.0312; Fig. 7B). The total signal frequency was also elevated (Student’s t-test, t = 6.045, df = 5, *p* = 0.0018; Fig. 7C). Analysis of spectral relative power revealed a significant reduction in low-frequency activity: delta (Student’s t-test, t = 3.630, df = 5, *p* = 0.0151; Fig. 7D) and theta waves (Student’s t-test, t = 3.639, df = 5, *p* = 0.0149; Fig. 7E). In contrast, high-frequency alpha (Student’s t-test, t = 3.394, df = 5, *p* = 0.0194; Fig. 7F) and beta waves (Student’s t-test, t = 6.580, df = 5, *p* = 0.0012; Fig. 7G) were significantly increased, while gamma activity remained unchanged (Student’s t-test, t = 0.6334, df = 5, *p* = 0.5543; Fig. 7H).

Together, these results indicate that hippocampal electrical activity is markedly altered during the 4-h rest period following subchronic SR, with a shift from low-frequency (delta/theta) to higher-frequency (alpha/beta) oscillations.

### Explicit Memory is Impaired After 4 h of Rest Following 10 Days of SR

Having observed that hippocampal CA1 electrical activity was altered during the 4-h rest opportunity, we hypothesized that SR animals would exhibit deficits in explicit memory after this period. To test this, we evaluated memory performance using the novel object recognition test (NORT) and the object location test (OLT).

**Fig. 8:**
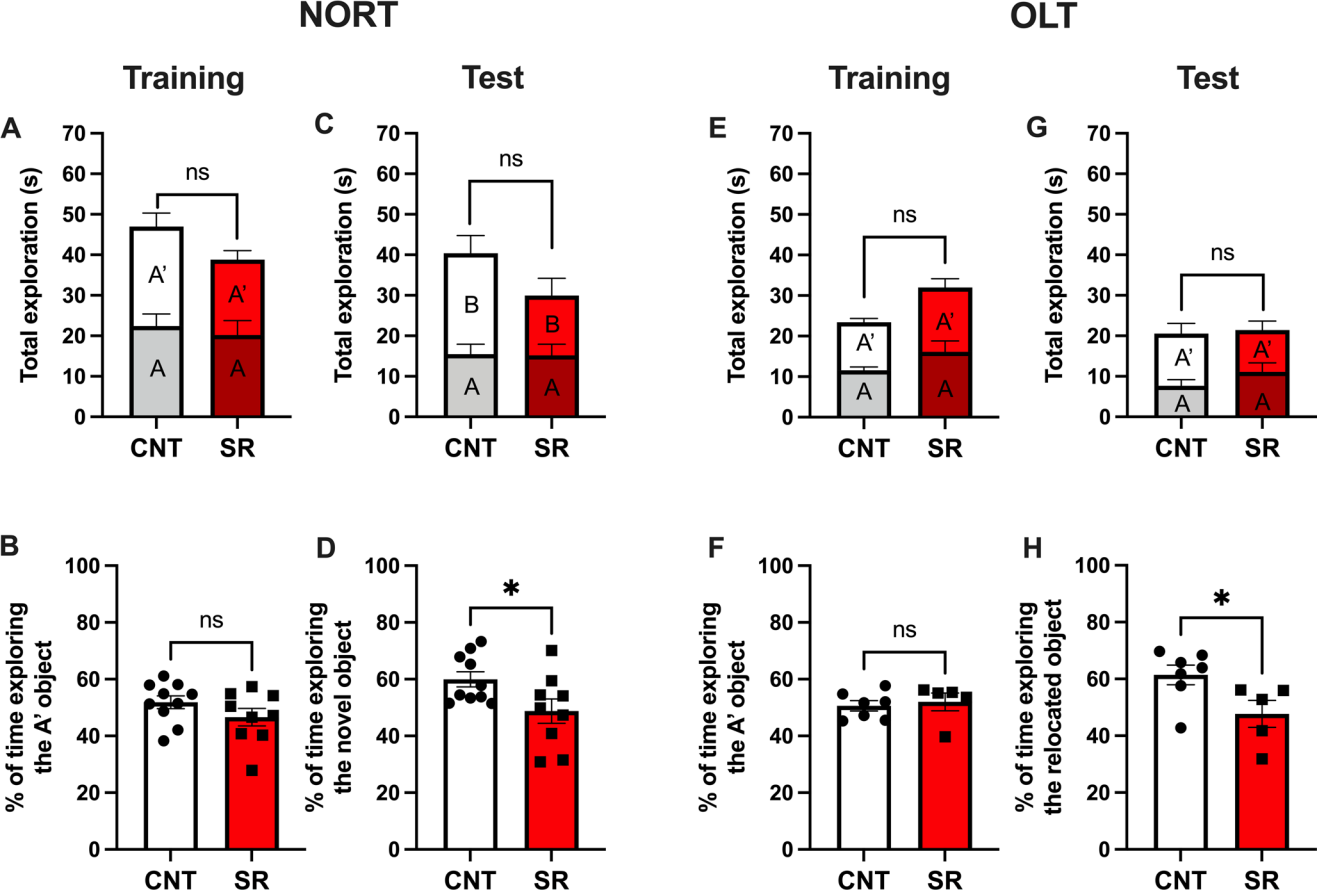
Explicit memory is impaired after 4 h of recovery following 10 days of sleep restriction. **A** Total exploration time during the training trial in the novel object recognition test (NORT) for control (CNT, white bars) and sleep-restricted (SR, red bars) animals. **B** Percentage of exploration time of object A’ during the training trial. **C** Total exploration time during the testing trial. **D** Percentage of exploration time of the novel object B during the testing trial. **E** Total exploration time during the training trial of the object location test (OLT) for CNT and SR animals. **F** Percentage of exploration time of object A′ during the training trial. **G** Total exploration time during the testing trial. **H** Percentage of exploration time of the relocated object A’ during the testing trial. Results are expressed as mean ± SEM. Statistical analysis was performed with unpaired Student’s t-tests. Asterisks indicate statistical significance: *p < 0.05

During NORT training, CNT and SR animals showed similar total exploration times for objects A and A’ (Student’s t-test, t = 1.092, df = 16, *p* = 0.2910; Fig. 8A) and no differences in the percentage of exploration of object A’ (Student’s t-test, t = 1.383, df = 17, *p* = 0.1845; Fig. 8B). This indicates that both groups explored the objects equally during training and that neither object was preferred.

One hour after training, during the test phase, both groups displayed comparable total exploration times (Student’s t-test, t = 1.122, df = 17, *p* = 0.2775; Fig. 8C). However, SR animals spent significantly less time exploring the novel object (Student’s t-test, t = 2.269, df = 17, *p* = 0.0366; Fig. 8D), indicating impaired short-term recognition memory formation.

In the OLT training session, no differences were observed between groups in total exploration time (Student’s t-test, t = 2.063, df = 10, p = 0.0661; Fig. 8E) or in the percentage of exploration of object A’ (Student’s t-test, t = 0.4138, df = 10, *p* = 0.6878; Fig. 8F). Similarly, during the OLT test trial, total exploration times did not differ (Student’s t-test, t = 0.1569, df = 10, *p* = 0.8785; Fig. 8G). In contrast, when analyzing exploration time for the relocated object A’, SR rats spent significantly less time exploring it compared to controls (Student’s t-test, t = 2.398, df = 10, *p* = 0.0374; Fig. 8H), indicating impaired contextual memory.

Importantly, neither total exploration times during training and testing nor corticosterone levels (Figure S6) differed between groups, indicating that reduced exploration of the novel or relocated object in SR animals was not attributable to decreased arousal, sleepiness, or stress.

## Discussion

The origins of SR-induced alterations in hippocampal neural network activity and how they relate to cognitive impairments observed after prolonged sleep disturbances remain poorly understood. In the present work, we found that the hippocampus of rats subjected to 10 days of SR using the MMPM protocol presents microglial alterations that persist after 4 h of sleep opportunity. In addition, the expression of synaptophysin, a presynaptic protein, and the ERK phosphorylation ratio, both involved in hippocampal long-term potentiation, were impaired after a short resting period. These results may be associated with alterations in hippocampal electrical activity during the last resting period of the 10-day SR protocol.

### Hippocampal-Dependent Memory is Affected by SR

The detrimental effects of sleep restriction have been widely studied [37], whereas the recovery process has received far less attention.

In SR rats, BBB permeability is impaired in areas such as the insular cortex, hippocampus, hypothalamus, basal ganglia, and cerebellum after 10 days of SR. A brief 2 h sleep recovery period restored BBB function in the cingulate gyrus, parietal and insular cortices, hypothalamus, basal nuclei, amygdala, thalamus, septal, and preoptic areas [18]. However, other regions including the hippocampus, rhinal, somatosensory, motor, auditory, orbitofrontal, and retro-splenial cortices did not recover [18].

Furthermore, a meta-analysis of sleep-deprived mice indicated that 3 h of sleep recovery is not enough to restore basal hippocampal gene expression [19]. In humans, two days of sleep recovery after one night of acute sleep deprivation did not restore episodic memory, despite hippocampal connectivity returning to baseline [15].

Another study tracked the effects of early-life SR induced by the MMPM, 20 h SR per day for 14 days beginning at postnatal day (PND) 28, observed impairments in long-term, but not short-term, memory at PND 42. This effect persisted until adulthood (PND 85) and was associated with reduced hippocampal neurogenesis and significantly less astrocytic aquaporin-4 (AQP4) immunoreactivity [38].

Some of the similarities among the above mentioned studies include alterations in neuronal counts and neurogenesis, reductions in the expression of synaptic proteins, and decreased performance in tests that evaluate learning and memory. Taken together, these data suggest that SR induces effects on memory that might persist over unknown time periods.

One challenge for comparing the available data is that each study employs different animal models, SR methods, durations, times of day, and moments of cognitive and mechanistic evaluations [39]. These differences complicate cross-study comparisons. Sequential evaluations at multiple time points following SR protocols may be necessary to determine the mechanisms involved in SR-induced cognitive impairments and their short- and long-term consequences.

### Microglial Function is Altered After SR

Microglial physiological function, morphology, and dynamics are regulated by spontaneous and evoked neuronal activity and vice versa. Studies have found that sleep modulates microglial morphodynamics through contact-dependent interactions such as CX3CR1, while Gi microglial signaling mediated by P2Y12 receptors regulates sleep architecture [40, 41]. In the present study, we observed that after 10 days of SR, hippocampal microglia showed significantly fewer branches. This decrease persisted after 4 h of sleep opportunity, indicating that this resting period is not sufficient to normalize microglial morphology.

A less ramified microglial profile is associated with phagocytosis [42–46]. Previous studies have shown that 24 h after SR, mice exhibit increased hippocampal microglial cell number, upregulated vesicular CD68 and C3aR, and decreased Syn and PSD-95 co-localization in CA1, which indicates reduced synaptic contacts due to increased phagocytic microglial activity [17]. In our study, IBA-1 IR and IBA-1 + cells did not remain elevated after a 4-h resting opportunity; instead, we observed a reduction in ramification of IBA-1 + cells, indicating a shift toward a reactive phenotype. Furthermore, we observed a significant decrease in both CA1 and CA3 Syn immunoreactivity immediately after SR, which persisted after 4 h of rest. We did not assess whether SR microglia contained Syn engulfments; however, the observed reduction in Syn could be associated with increased microglial phagocytic activity. Future studies should assess whether microglial morphological changes after 4 h of rest are associated with increased phagocytic activity and contribute to Syn reduction.

A less ramified phenotype has also been associated with microglial-mediated neuroinflammation [47]. Neuroinflammatory models such as brain infarcts or intracerebroventricular lipopolysaccharide injections are known to induce a less ramified microglial morphology [48, 49]. Similarly, studies have shown reduced ramification length of cortical microglia in SR mice [50]. Furthermore, IL-1β, IL-6, and TNF-α increase in the hippocampus and/or prefrontal cortex of SR mice [51, 52], indicating that the microglial morphological changes observed in the present study may be associated with a pro-inflammatory profile.

Microglial morphology has also been associated with neuronal oscillations. In mice subjected to extended waking, keeping animals awake for 3 h during their usual resting phase enhanced cortical theta/gamma frequency, which was associated with increased alertness and microglial ramification in the somatosensory cortex and hippocampus. When waking was extended to 9 h, microglial ramifications decreased in both regions, which was associated with a gradual increase in slow wave activity (delta) [26]. These results in two acute paradigms are consistent with our data, which show a decrease in ramification after prolonged SR associated with reduced hippocampal theta frequency. Although the two studies differed in duration and in the signals recorded (cortical EEGs vs. hippocampal LFPs), both converge on the association between microglial ramification and theta activity, indicating that this rhythm may be important for maintaining microglial homeostasis, or conversely, that microglial state may influence theta activity.

Microglial P2Y12–Gi activation promotes NREM sleep by reducing norepinephrine cortical transmission [41]. As mentioned before, the loss of microglial homeostatic function is observed during SR; therefore, we hypothesize that changes in hippocampal electrical activity after SR could be associated with changes in microglial activity, specifically with Gi activation. Future studies should assess whether hippocampal microglial Gi signaling is altered during SR and whether its restoration could prevent the cognitive impairments observed.

### Hippocampal Electrical Activity is Altered After SR

Hippocampal LFPs are characterized by slow rhythmic activity, mainly represented by the theta (4–8 Hz) rhythm that organizes neuronal firing rate and is associated with the processing of environmental signals and memory encoding [53–55]. Previous studies have determined that theta rhythm in the entorhinal–CA1 pathway is necessary for memory encoding, while theta rhythm in the Schaffer collateral pathway underlies memory consolidation and retrieval [53]. In the present study, SR animals were unable to recognize the novel object even when they were allowed to sleep for 4 h following the MMPM protocol. This effect could be associated with the reduced amount of theta rhythm observed during the 4 h of rest.

In addition, hippocampal gamma frequency is important for information transfer between CA3 and CA1 and plays a role in memory consolidation and retrieval [53, 56]. The CA3–CA1 pathway displays distinctive theta–gamma coupling during exploratory and memory-guided behaviors [35, 57]; disrupting hippocampal theta oscillation during REM sleep after learning prevents memory consolidation [18]. In the present study, we did not observe changes in gamma relative power during the 4 h resting opportunity. Although there are no studies directly addressing whether theta–gamma coupling occurs during sleep, future studies should assess whether SR interferes with hippocampal theta–gamma coupling during exploratory tasks and across sleep stages.

Furthermore, stimulation of the entorhinal cortex (EC) at low (1 Hz) or high (20 Hz) frequencies fails to elicit long-term potentiation (LTP) in CA1 compared with theta frequency stimulation (5 Hz) [58]. This is because the connections between the DG and CA3 serve as a filter, preventing low and high frequencies from reaching CA1 [59]. Here, we observed that SR increased high-frequency alpha and beta rhythms (8–12 Hz and 12–30 Hz, respectively) in the hippocampus during the resting opportunity. This shift from theta to higher-frequency activity in the hippocampus may contribute to decreased performance during behavioral tests. Indeed, hippocampal theta frequency has been shown to induce LTP in CA1 [60], and optogenetic inhibition of hippocampal theta during REM sleep impairs subsequent performance in the NORT [36]. Future studies should investigate whether reduced theta rhythm after SR is responsible for the associated memory impairments and altered Syn expression and ERK activation.

The homeostatic regulation of sleep increases sleep pressure in proportion to the duration of wakefulness; when sleep time is reduced, the organism compensates through a process known as sleep rebound [61]. In rodents, EEG studies have shown an immediate compensatory increase in cortical NREM delta power and in the percentage of NREM sleep following acute total sleep deprivation [61–63]. However, this compensatory response is no longer evident under repeated sleep restriction. Kim et al. reported that after 5 consecutive days of sleep restriction (20 h SR followed by 4 h of rest each day), mice failed to exhibit a compensatory increase in cortical NREM delta power. Moreover, cortical NREM delta power remained below baseline levels for two additional days after the restriction protocol ended [64].

At the hippocampal level, theta rhythm is normally up-regulated during NREM [65], while theta oscillations during REM are critical for both memory consolidation and selective forgetting [66]. In a related study using 6 days of sleep deprivation, hippocampal EEG revealed that training shifted activity from irregular mixed waves to rhythmic patterns; however, the proportion of theta (4–8 Hz) increased only transiently in the SR group (restricted to the first two training days), whereas control animals displayed a sustained theta enhancement while awake [67]. To our knowledge, no studies have explicitly measured or documented delta/theta rebound in hippocampal LFP recordings during NREM or REM following sleep restriction. However, our data show no increase in delta or theta power after 10 days of SR, suggesting that repeated sleep loss disrupts compensatory rebound mechanisms both during learning and across sleep states. Importantly, prospective studies will be needed to determine whether subchronic protocols similar to ours may also lead to persistent effects comparable to those described in chronic paradigms, such as the study by Owen et al. [31], in which long-term spatial memory deficits and hippocampal pathology were evident months after the restriction period.

### Syn and ERK Changes During SR

Syn is a presynaptic vesicle protein involved in synaptic transmission, and its expression positively correlates with learning and memory performance [68–70]. Processes such as LTP are associated with increased Syn expression and ERK activity [71, 72]. A decrease in hippocampal ERK activity and Syn expression has been reported in Ndufs4 knockout mice [73]. These findings indicate that ERK activity and Syn expression are closely linked. In our study, we observed a reduction in Syn and an increase in pERK after a 4 h resting opportunity, indicating changes in synaptic transmission.

ERK activation is essential for the induction and maintenance of LTP by modulating AMPA receptor trafficking [72]. Moreover, administration of U0126, an ERK inhibitor, impairs hippocampal LTP induced by 5 Hz (theta rhythm) stimulation [33]. This suggests that hippocampal LTP induced by theta rhythm is ERK-dependent. Therefore, the increase in pERK after SR may be associated with processes other than LTP.

The ERK pathway also modulates the sleep–wake cycle. Specific ERK1/2 deletion or pharmacological inhibition increases wakefulness after 6 h of sleep deprivation in mice [74]. This suggests that ERK phosphorylation contributes to sleep pressure, the biological drive to sleep that accumulates with time awake. In the present study, pERK levels significantly increased after 4 h of rest. This could represent a compensatory effect of SR that enhances sleep pressure; however, it does not explain why pERK was not increased immediately after restriction.

The effects of SR on ERK modulation appear to depend on protocol duration. Acute SR/SD in Sprague–Dawley rats reduces hippocampal ERK phosphorylation [75–77]. These results contrast with our data, since we observed the opposite effect. This discrepancy may be explained by the acute nature of the protocols used by Guan et al., Ravassard et al., and Wang et al., compared with the subchronic character of ours. This interpretation is further supported by data from long-term SR protocols lasting up to 6 months, in which ERK phosphorylation is increased in the cerebellum [78]. In addition, the hippocampus of epileptic mice also presents elevated pERK [79, 80]. Collectively, these interventions involve profound homeostatic alterations, including upregulation of inflammatory mediators in the CNS [81, 82], indicating that pERK increases in response to either prolonged or extreme physiological challenges.

Activation of the ERK pathway is also required to initiate the inflammatory cascade in glial cells. Microglia isolated from 5xFAD mice show increased ERK phosphorylation [83], which is a critical regulator of microglial pro-inflammatory activation [84]. Moreover, ERK activation enhances microglial migration and proliferation in a rat model of neuropathic pain [85], and stimulation of this pathway increases phagocytic activity in cultured BV2 cells [86]. As previously noted, SR protocols promote pro-inflammatory processes in the CNS; therefore, the increase in pERK observed here may also be related to the up-regulation of inflammatory pathways.

### Limitations of the Study

While our findings provide new insights into the effects of SR, a number of limitations remain. First, only male rats were included, since our previous work and direct references were performed in males, which facilitated comparisons across studies. However, we recognize that female subjects have been historically underrepresented in sleep research, and it is imperative to determine whether SR and recovery differentially affect the female hippocampus. Parallel studies in our group are currently being performed in females to directly address this question.

Second, we did not record EMG signals due to the high risk of infection during the MMPM protocol and the technical complexity of maintaining electrodes in a water-based paradigm. As a result, we could not formally score sleep stages to directly relate hippocampal rhythms to the different sleep phases, and our conclusions are therefore limited to hippocampal LFP recordings obtained during the recovery period.

Third, hippocampal LFPs were not recorded along the 20 h restriction phases, but only during the last 4 h of each cycle, as the MMPM protocol required animals to remain in the restriction cage in pairs to minimize isolation stress. However, pair housing introduced the possibility of electrode displacement due to animal interactions. Consequently, hippocampal network dynamics throughout the restriction phases could not be evaluated.

Fourth, we restricted our analysis to the immediate 4-h resting period following 10 days of SR; therefore, we cannot determine whether the observed alterations persist, normalize, or progress at later time points. Fifth, although we observed correlations between hippocampal electrical activity, microglial morphology, Syn expression, and ERK phosphorylation, the study design does not allow us to establish causal relationships. Finally, our mechanistic scope was limited: we did not directly assess microglial phagocytosis, inflammatory mediator levels, or additional signaling pathways that may contribute to the observed outcomes. Likewise, the analysis was restricted to Syn as a presynaptic marker and ERK phosphorylation as a molecular correlate of plasticity.

Future studies should aim to describe additional parameters, including other synaptic proteins, inflammatory mediators, and extended LFP and behavioral analyses, to provide a more comprehensive understanding of hippocampal alterations induced by sleep restriction.

## Conclusion

In the present work, we observed that hippocampal electrical activity is altered after 10 days of sleep restriction. Analysis of hippocampal LFPs during the immediate resting opportunity revealed increased alpha and beta rhythms together with decreased delta and theta waves compared to non-restricted rats, indicating that the long-term effects of SR may be associated with sustained deregulation of hippocampal neuronal and glial circuits. This study also demonstrates the persistence of alterations in microglial morphology, reduced Syn immunoreactivity, and an increased pERK/ERK ratio after resting, which may contribute to the cognitive impairments observed.

Although long-term effects of SR in the hippocampus have been previously reported, our study provides evidence linking these changes with hippocampal electrical activity. We cannot establish a causal relationship between hippocampal oscillations and SR-induced cognitive deficits; however, we identify potential cellular and molecular targets known to influence hippocampal activity that should be explored in future studies to prevent both short- and long-term consequences of SR. This is particularly relevant given that chronic sleep reduction in humans has been associated with an increased risk of neurodegenerative disorders such as Alzheimer’s disease, a condition promoted by microglial pro-inflammatory activity and neuronal degeneration.

## Supplementary Information

Below is the link to the electronic supplementary material.

Supplementary material 1 (DOCX 54301.9 kb)

## Data Availability

No datasets were generated or analysed during the current study.
