# Supplementary material for: A Sleeping Opportunity Does Not Restore Hippocampal Alterations Induced by 10 Days of Sleep Restriction in Rats
Source: Neurochem Res. 2025 Sep 29;50(5):311. doi: 10.1007/s11064-025-04561-1 (PMC12479644; doi:10.1007/s11064-025-04561-1)
Supplement: Supplementary file 1 — Supplementary material 1 (DOCX 54301.9 kb) [file 11064_2025_4561_MOESM1_ESM.docx]

**Supplementary information**

**Figure S1**


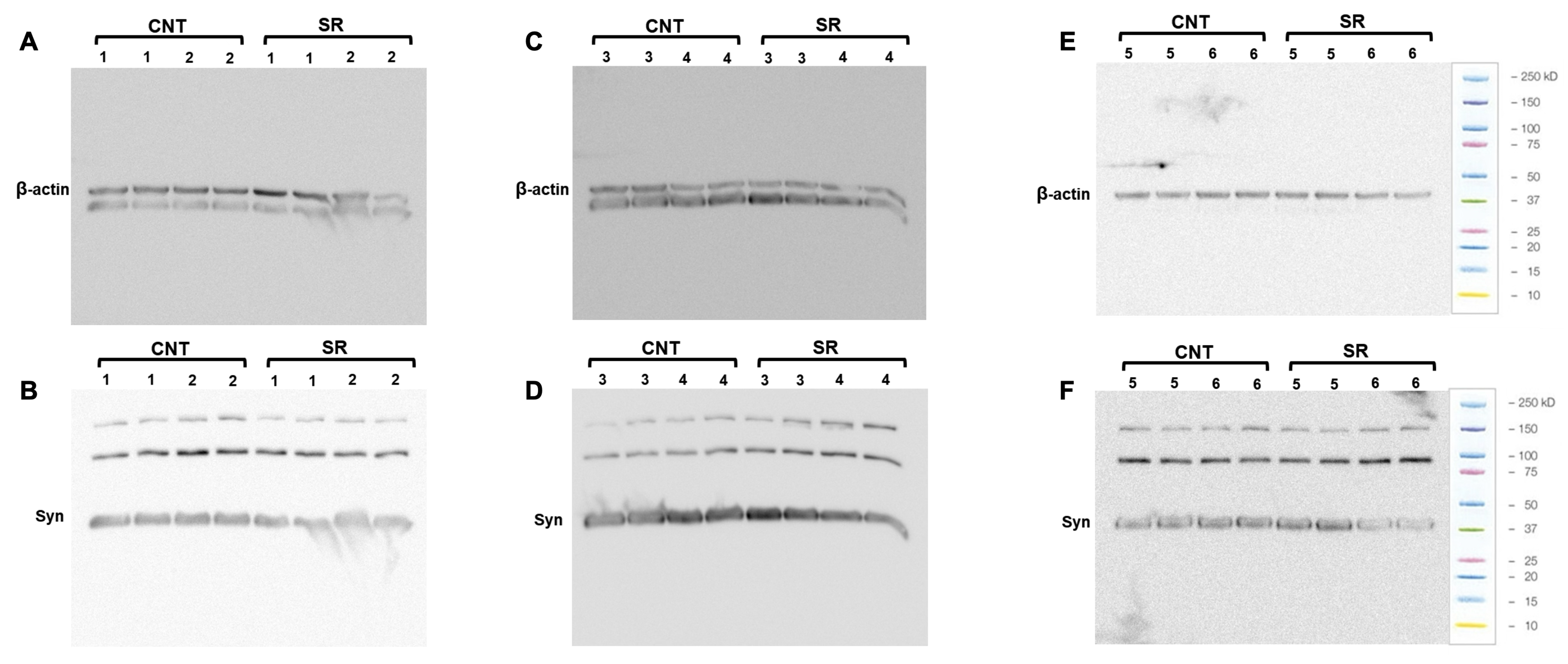


**Figure S1. Full-length blots of β-actin and Synaptophysin detection in the hippocampus immediately after 10 days of sleep restriction. (**A, C, and E) β-actin blots (40 kDa) of the control (CNT) and sleep-restricted (SR) animals. (B, D, and F) Synaptophysin (Syn, 38 kDa) of the CNT and SR animals. The number of each animal and the group to which it belongs is indicated at the top of each blot. Samples were loaded in duplicate.

**Figure S2**


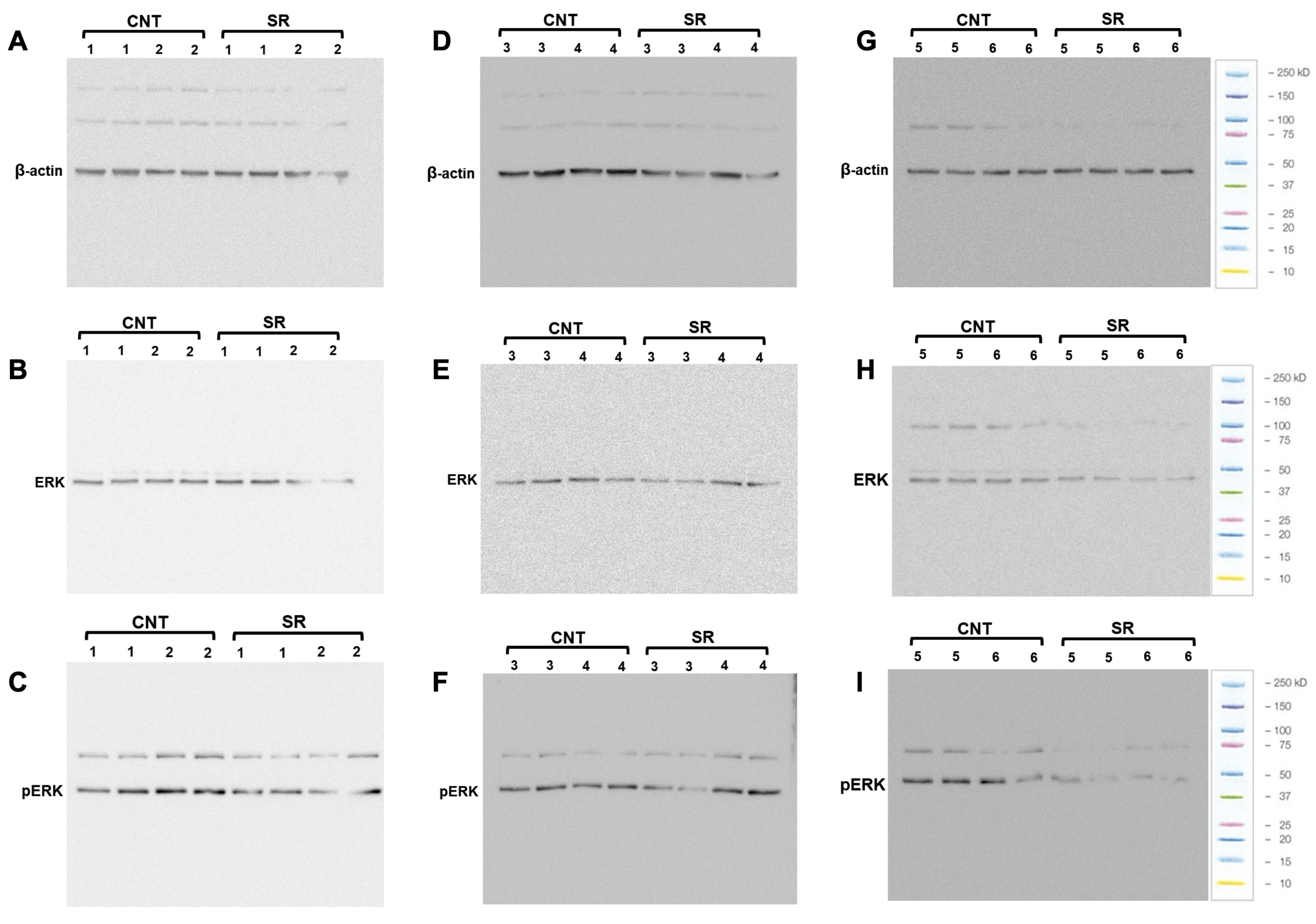


**Figure S2. Full-length Western blots of β-actin, ERK, and phosphorylated ERK in the hippocampus immediately after 10 days of sleep restriction.** (A, D, G) Full-length β-actin blots (40 kDa) from control (CNT) and sleep-restricted (SR) animals. (B, E, H) Full-length total ERK blots (44 kDa) from CNT and SR animals. (C, F, I) Full-length phosphorylated ERK (pERK, 44 kDa) blots from CNT and SR animals. The number of each animal and its group is indicated at the top of each blot. All samples were loaded in duplicate.

**Figure S3**


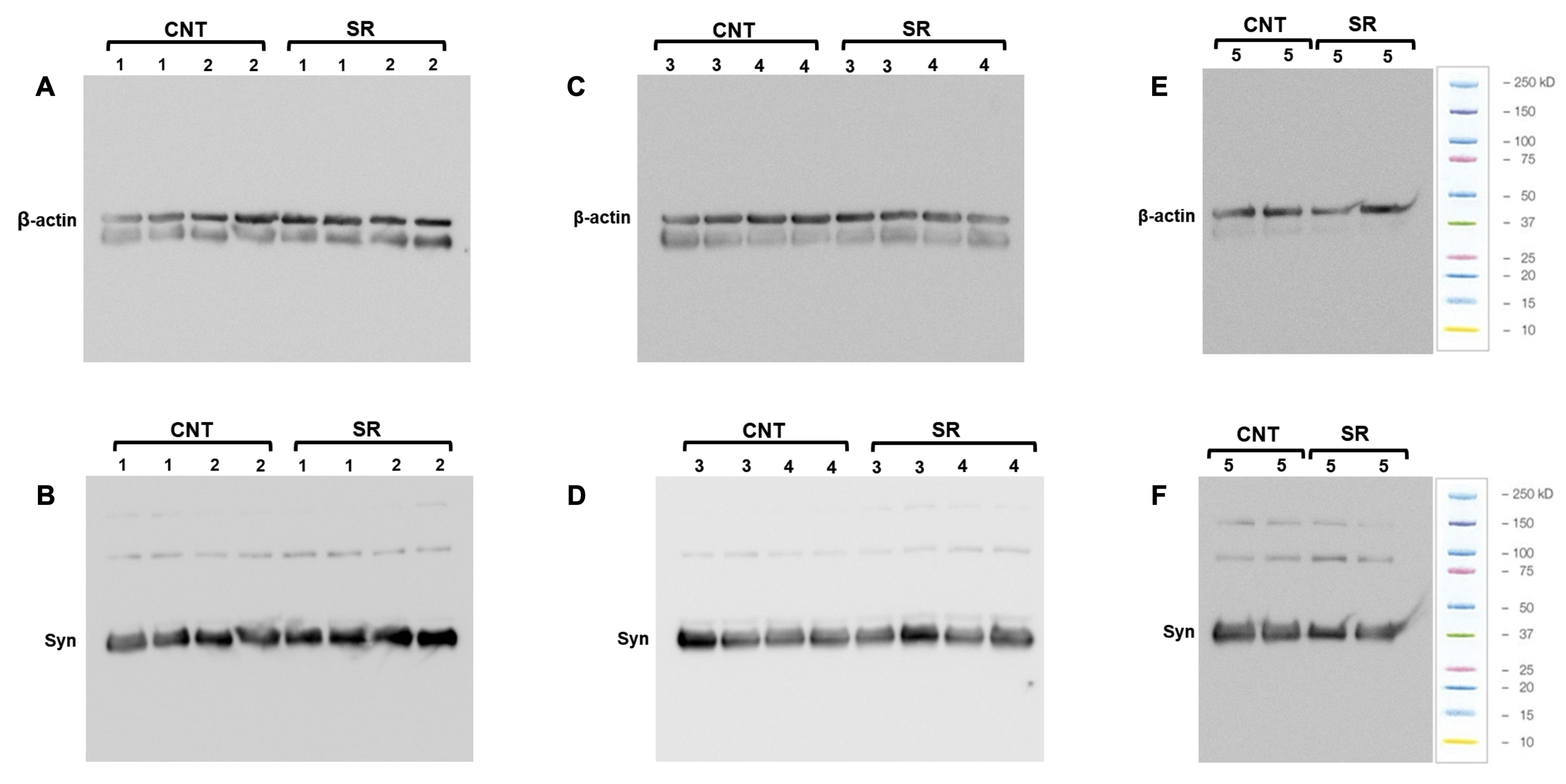


**Figure S3. Full-length Western blots of β-actin and synaptophysin in the hippocampus after 4 h of recovery following sleep restriction.** (A, C, E) Full-length β-actin blots (40 kDa) from control (CNT) and sleep-restricted (SR) animals. (B, D, F) Full-length synaptophysin (Syn, 38 kDa) blots from CNT and SR animals. The number of each animal and its group is indicated at the top of each blot. All samples were loaded in duplicate.

**Figure S4**


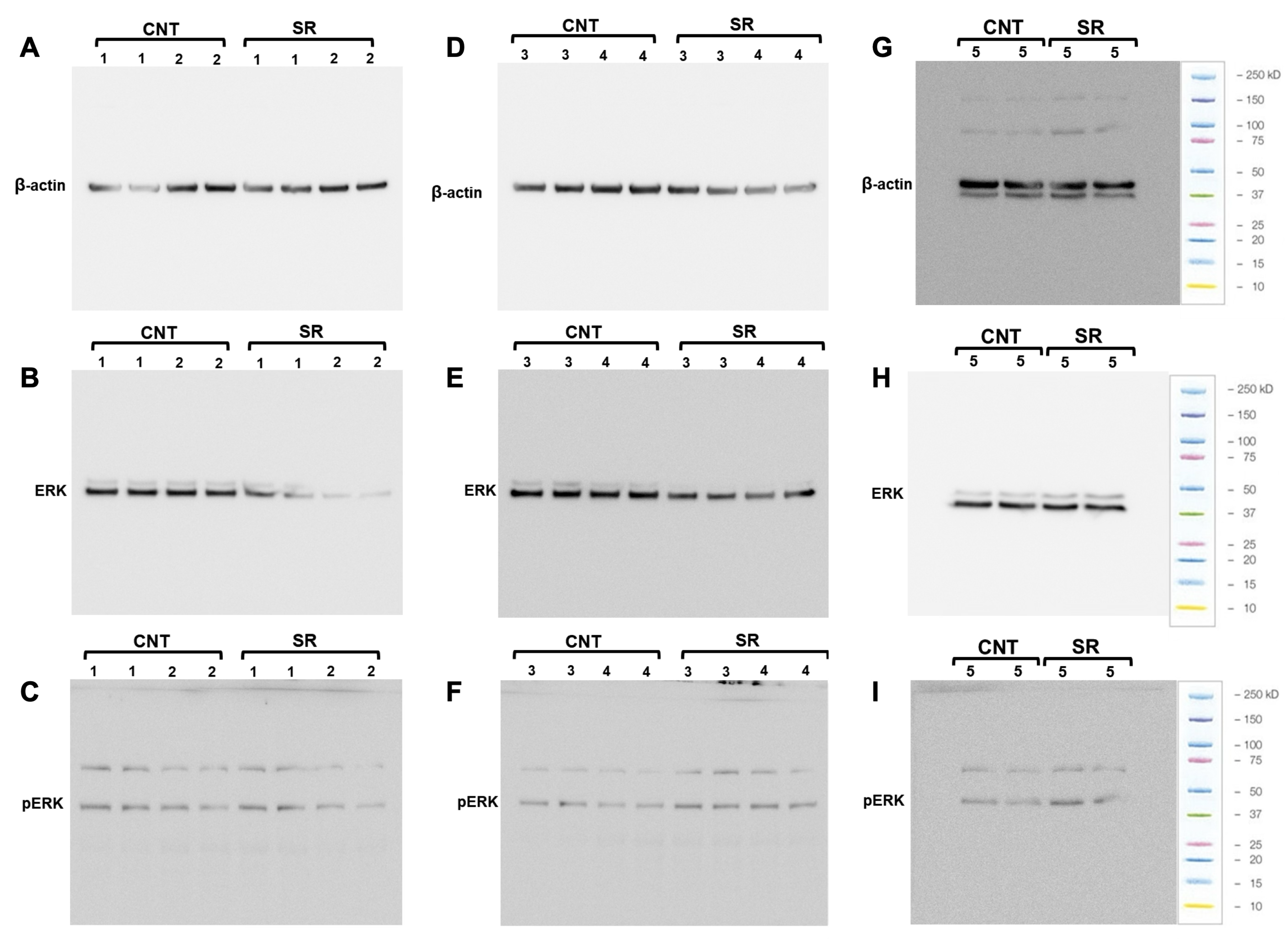


**Figure S4. Full-length Western blots of β-actin, ERK, and phosphorylated ERK in the hippocampus after 4 h of recovery following sleep restriction.** (A, D, G) Full-length β-actin blots (40 kDa) from control (CNT) and sleep-restricted (SR) animals. (B, E, H) Full-length total ERK blots (44 kDa) from CNT and SR animals. (C, F, I) Full-length phosphorylated ERK (pERK, 44 kDa) blots from CNT and SR animals. The number of each animal and its group is indicated at the top of each blot. All samples were loaded in duplicate.

**Figure S5**


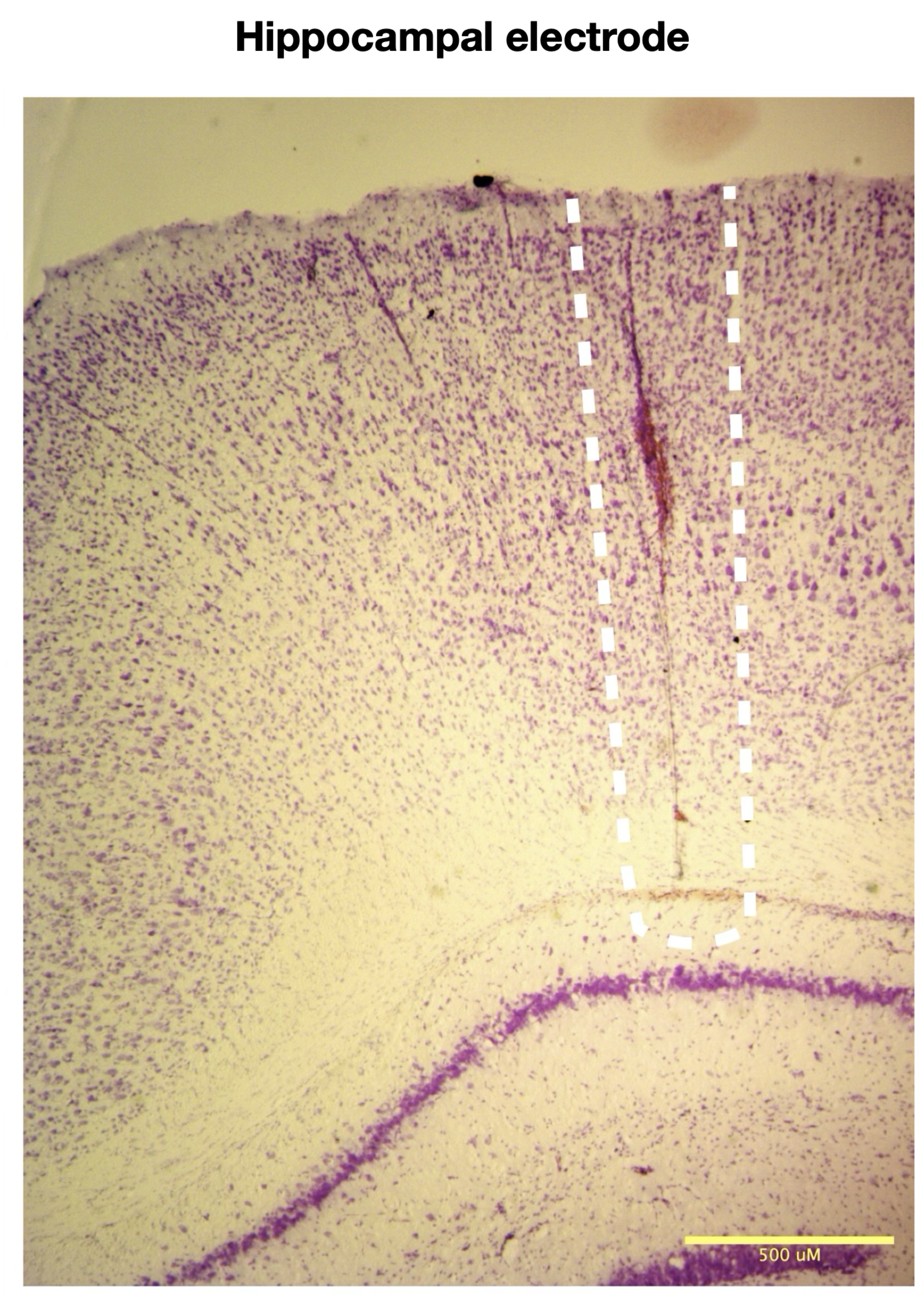


**Figure S5. Electrode implantation sites in the hippocampus.** Representative Nissl-stained microphotographs showing electrode tracks (dashed white lines) in the CA1 region of the left dorsal hippocampus.

**Figure S6
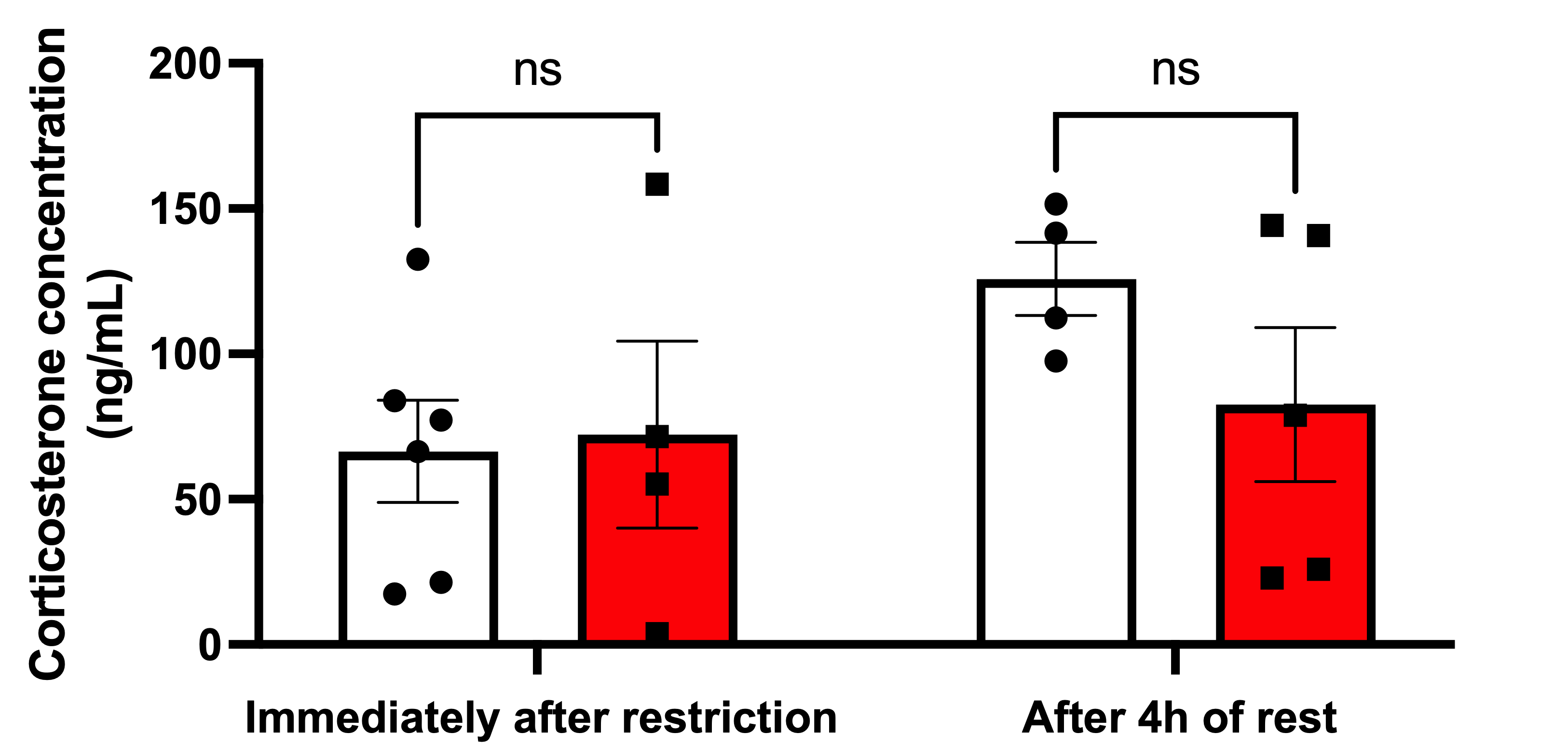
**

**Figure S6. Blood corticosterone levels in control and sleep-restricted rats.** Plasma corticosterone concentrations measured by radioimmunoassay in control (CNT, white bars) and sleep-restricted (SR, red bars) animals immediately after the 10-day SR protocol and after 4 h of recovery. Statistical analysis was performed with two-way ANOVA (sleep condition: F[1,15]=0.6477). No significant differences were observed either immediately after restriction (p = 0.8605) or after 4 h of rest (p = 0.2170).
